# *Brassica napus* L. cultivars show a broad variability in their morphology, physiology and metabolite levels in response to sulfur limitations and to pathogen attack

**DOI:** 10.3389/fpls.2015.00009

**Published:** 2015-02-02

**Authors:** Annekathrin Weese, Philip Pallmann, Jutta Papenbrock, Anja Riemenschneider

**Affiliations:** ^1^Institute of Botany, Leibniz University HannoverHannover, Germany; ^2^Institute of Biostatistics, Leibniz University HannoverHannover, Germany

**Keywords:** canola, diurnal rhythm, elemental sulfur, metabolites, *Verticillium longisporum*

## Abstract

Under adequate sulfur supply, plants accumulate sulfate in the vacuoles and use sulfur-containing metabolites as storage compounds. Under sulfur-limiting conditions, these pools of stored sulfur-compounds are depleted in order to balance the nitrogen to sulfur ratio for protein synthesis. Stress conditions like sulfur limitation and/or pathogen attack induce changes in the sulfate pool and the levels of sulfur-containing metabolites, which often depend on the ecotypes or cultivars. We are interested in investigating the influence of the genetic background of canola (*Brassica napus*) cultivars in sulfur-limiting conditions on the resistance against *Verticillium longisporum*. Therefore, four commercially available *B. napus* cultivars were analyzed. These high-performing cultivars differ in some characteristics described in their cultivar pass, such as several agronomic traits, differences in the size of the root system, and resistance to certain pathogens, such as *Phoma* and *Verticillium*. The objectives of the study were to examine and explore the patterns of morphological, physiological and metabolic diversity in these *B. napus* cultivars at different sulfur concentrations and in the context of plant defense. Results indicate that the root systems are influenced differently by sulfur deficiency in the cultivars. Total root dry mass and length of root hairs differ not only among the cultivars but also vary in their reaction to sulfur limitation and pathogen attack. As a sensitive indicator of stress, several parameters of photosynthetic activity determined by PAM imaging showed a broad variability among the treatments. These results were supported by thermographic analysis. Levels of sulfur-containing metabolites also showed large variations. The data were interrelated to predict the specific behavior during sulfur limitation and/or pathogen attack. Advice for farming are discussed.

## Introduction

Oilseed rape or canola (*Brassica napus* L.) belongs to the Brassicaceae family. Oilseed rape is used for the production of green fuel, human consumption, as animal feed, in the chemical and pharmaceutical industry (Friedt and Snowdon, 2009), and has an enormous economical importance for many farmers in Europe (European Commission Eurostat, 2014).^1^ Compared with crops such as wheat, soybean and rice, which have a long history of evolution and domestication, rapeseed is a recently domesticated species. It possibly arose as a result of interspecific hybridizations and genome doubling between diploid genotypes of turnip rape (*Brassica rapa*, 2*n* = 2 × 10 = 20, genome AA) and cabbage (*Brassica oleracea*, 2*n* = 2 × 9 = 18, genome CC) that occurred spontaneously during medieval times or earlier (Iñiguez-Luy and Federico, 2011). Vollmann and Rajcan (2009) summarized that in general, the breeding of oil crops is a more complex undertaking than breeding of cereals or legumes because most of the oil crops are dual- or multi-purpose crops. Often simultaneous manipulations are required to create different characteristics of quality.

Oilseed rape has higher requirements for nitrogen, phosphorus and sulfur than cereals and other crops. *Brassica napus* plants need approximately 40–50 kg of nitrogen (30% more than wheat), 8 kg phosphorus and 10 kg sulfur per metric ton of grain produced. In fact, wheat needs 15–25 kg sulfur ha^−1^, whereas *B. napus* needs 30–50 kg sulfur ha^−1^ (Bloem and Haneklaus, 2002).

The high demand of sulfur supplementation in rapeseed occurred as a cause of sulfur-deficient soils and dramatic reduction in atmospheric deposition of sulfur in recent years due to enhanced emission controls (Dämmgen et al., 1998; Lewandowska and Sirko, 2008). This reduction has had significant impact on agriculture; most notably as oilseed rape has been exhibiting sulfur deficiency symptoms. (Schnug et al., 1995). Under sulfur limitation [for definitions of the sulfur status see Scherer (2001)], crops begin to develop sulfur deficiency symptoms such as reduced plant growth and chlorosis of the younger leaves (Grant and Kovar, 2012). Symptoms become visible in crop plants in the following order: first in oilseed rape, potato, sugar beet, beans, peas, cereals and finally in maize. The total sulfur concentration in tissues corresponding to the first appearance of deficiency symptoms is highest in oilseed rape (3.5 mg sulfur g^−1^ dry weight, DW) and lowest in the Gramineae (1.2 mg sulfur g^−1^ DW) (Haneklaus et al., 2007). Long term sulfur deficiency can lead to reduced yield and crop quality (Ahmad and Abdin, 2000; Scherer, 2001). An increase of diseases due to enhanced emission controls led to the hypothesis that there might be a relationship between the sulfur supply, the high sulfur demand of rape (Holmes, 1980), and defense mechanisms against fungal diseases. The hypothesis of sulfur-induced resistance (SIR) (Schnug et al., 1995) and a sulfur-enhanced defense (SED) (Kruse et al., 2007) was proposed.

In recent years, lab-scale experiments have produced substantial amounts of data supporting the conjecture that sulfur-containing compounds play a role in pathogen defense. *Arabidopsis thaliana* wild-type and knockout plants were used to investigate the role of cysteine in response to pathogen attack by using *Pseudomonas syringae* pv. *tomato, Botrytis cinerea* (Álvarez et al., 2011), and *Alternaria brassicicola* (Kruse et al., 2012). Cysteine is a precursor for essential vitamins, cofactors, and many defense compounds such as glucosinolates (GSL), thionins, or phytoalexins (Smith and Kirkegaard, 2002; Van Wees et al., 2003; Rausch and Wachter, 2005). In infected plants, the cysteine content decreased by 24–28% but a 14–15% increase in glutathione content was observed (Álvarez et al., 2011).

Williams et al. (2002) demonstrated that elemental sulfur was formed and accumulated in tomato plants in response to infection with *Verticillium dahliae*. In older leaves, the sulfate content increased after 14 days post-infection (dpi), indicating that sulfate levels in infected plants were dependent on the leaf age. Significant changes in cysteine levels of plants infected with *V. dahliae* were measured in the stem vascular tissue. The same was observed for the glutathione content in leaves. In the past, reduction of sulfur containing metabolites especially in young leaves was shown in *B. napus* under sulfur limitation (Blake-Kalff et al., 1998).

Field experiments with *B. napus* and *Pyrenopeziza brassicae* have shown an increase of the thiol concentration. Crops were able to react to a fungal infection and had a greater potential to release H_2_S, which is reflected by a positive correlation between L-cysteine desulfhydrase enzyme activity and fungal infection levels (Bloem et al., 2004). Consecutive greenhouse experiments have shown that already after one dpi, the H_2_S emission of plants grown under full sulfur supply increased strongly (Bloem et al., 2012).

Haneklaus et al. (2007) concluded that under sulfur deficiency, *B. napus* develops the most distinctive and most specific expression of phenotypic symptoms. No difference in the symptomatology of sulfur deficiency was observed in high and low containing GSL cultivars. Differences in their susceptibility against fungal infections were not unambiguously demonstrated so far.

Several winter rape cultivars with low erucic acid and low total GSL contents, <30 μmol g^−1^ defatted seed meal, were investigated to determine their contents of GSL in leaves and roots. Also in these organs, the GSL levels are rather low. In leaves, the concentrations of total GSL were about 3 μmol g^−1^ DM and in roots, about 18 μmol g^−1^ DM (Eberlein et al., 1998). In very recent high-performance cultivars, the GSL contents are even lower (see Table 1) and probably do not contribute much to the total sulfur amount in modern *B. napus* cultivars (Eberlein et al., 1998).

**Table 1:** **Summary of available data about the different cultivars**.

**Cultivar**	**Type**	**Vigor**	**Resistance to *Verticillium* wilt**	**Root system**	**Oil content**	**GSL content [μmol g^−**1**^ seed DM]**
Compass	H	+++	++++	++++	++++++	<18
Exocet	H	++++++	+++	++++++	++++	<25
Genie	H	++++++	++++	+++	+++++	<25
King10	L	++++++	++++++	−	++++++	<25

*H, hybrid; L, line-bred; +, intensity; −, no information. The intensity is rated based on parameter values found on the websites given in Materials and Methods (Range from + very low to ++++++ very high). DM, dry mass; GSL, glucosinolates*.

As mentioned before, yield reductions of *B. napus* is caused by not only a sulfur deficiency, but also a Verticillium infection (Dunker et al., 2008). The soil-borne vascular fungal pathogen *Verticillium longisporum* is one of the most important yield-minimizing pathogens of oilseed rape, and there has been no approved fungicide available until now (Heale and Karapapa, 1999; Friedt and Snowdon, 2009). In addition, the fungus survives in the soil for long periods through the production of microsclerotia. Therefore, selection of suitable resistant cultivars and optimized cultivation methods need to be developed. As was reported previously, many pathogens attack plants during dawn by dispersing their spores (Wang et al., 2011). However, there is no knowledge of exactly when *V. longisporum* attacks. Interestingly, expression studies of adenosine 5′-phosphosulfate reductase (APR) indicate that sulfur assimilation is controlled in a diurnal way (Kopriva et al., 1999).

Results from Burandt et al. (2001) indicate that the capability to use available soil sulfur is genetically controlled. Therefore, different high-performance cultivars currently cultivated in Europe were analyzed. The high-performance cultivar plants were compared at early stages of development under controlled conditions, irrespective of the final yield. (I) We were interested in the metabolic reaction to sulfur limitation and the reaction of *B. napus* cultivars to pathogen attack of *V. longisporum*. (II) To better understand the mechanisms of SED of the high-sulfur-demanding *B. napus* plants, the influence of time points during the day was analyzed. (III) The plants were comprehensively analyzed by measuring biometrical and physiological parameters, levels of several sulfur- and non-sulfur containing metabolites, and gene expression levels. After analyzing *B. napus* plants in an early stage of development, a recommendation for a promising cultivar to avoid yield loss is given.

## Materials and methods

### Plant material and growth conditions

#### Plant material

Winter oilseed rape seeds from the cultivars Compass, Exocet, Genie and King10 were obtained from the D^2^ eutsche Saatveredelung AG (DSV) (Lippstadt, Germany) (http://www.dsv-saaten.de/raps/winterraps/sorten). The most important traits are summarized in Table 1. Compass is an MSL-hybrid (MSL, Male Sterility Lembke), has an excellent resistance to lodging of insects and has been on the market in Germany since 2009. Exocet is an OGURA-hybrid, which has been introduced to the market in 2005 (OGURA, expressed as cytoplasmatic male sterility, originated from *Raphanus sativus*) (Ogura, 1968). It has a high grain yield potential and excellent resistance against blackleg. Genie is an MSL-hybrid and has been introduced in 2010. It is very vital and has an extremely low temperature resistance. In addition to the three hybrid varieties, we have chosen line King10 that is a line-bred cultivar and was market-authorized in 2009. The yield of line King10 is comparable with new high-performance hybrids. The three varieties and the line will be summed up as cultivars. More information about the cultivars can be found on several websites (www.rapool.de^3^, www.roth-agrar.de^4^ and www.dsv-saaten.de/raps/winterraps/sorten).

#### Plant growth

For infection experiments, 30 seeds per plate of all four cultivars were sterilized and placed on plates containing solidified Blake-Kalff medium (Blake-Kalff et al., 1998) with 1 mM MgSO_4_. After 7 day of germination in a climatic chamber [22°C, 70% humidity, 12 h light/12 h dark, 480 μmol m^−2^ s^−1^(lamp type CMT 360LS/W/BH-E40, Eye Lighting Europe Ltd, Uxbridge, UK)], 45 seedlings were mock-inoculated with water or root dip-inoculated for 30 min (the production of spores is described below) and transferred in pots (8 cm diameter) filled with sand (0–2 mm grain size, Hornbach, Hannover, Germany). After pre-experiments reclined to experiments of Blake-Kalff et al. (1998), three different sulfur regimes with respect to sulfur concentration and volume of nutrient solution per week were chosen. As a control, one third of the pots were irrigated with Blake-Kalff medium containing 1 mM MgSO_4_ (full sulfur supply, optimal growth conditions), the other pots were treated with Blake-Kalff medium containing 0.025 mM (moderate sulfur limitation) or 0.010 mM MgSO_4_ (severe sulfur limitation) and additional MgCl_2_. Each pot was watered with 150 ml of a nutrient solution weekly. After 14 dpi, the leaves and the stems of three plants of each treatment were harvested every 4 h, beginning 1 h before light was switched on (labeled 0, 4, 8, 12, and 16 h in the graphs). Plant material was pooled and directly frozen in liquid nitrogen for further analyses.

#### Pathogen cultivation

For the production of *Verticillium longisporum* spores, 500 μl of a frozen spore culture (isolate VL43, Enyck et al., 2009) was cultivated in 500 ml potato dextrose liquid medium (Difco PDB, Becton, Dickinson and Company, New Jersey, USA) in 1 L flasks. The flasks were incubated at 23°C in a rotary incubator at 150 rpm in darkness for 2 weeks until a dense spore suspension was produced. The concentration of the filtered spores per ml suspension was determined using a Thoma chamber and diluted with sterile water (pH 7.0) to 1^*^10^6^ spores per ml.

### Thermographic analysis

The evaporative cooling as water is lost through stomata is an important component of the local leaf energy balance. Thus, leaf temperature can provide a sensitive indicator of leaf conductance to water vapor (Jones, 2007). In several studies, leaf temperature was used as an indicator of water stress, salt stress and nutrition deficiency induced stress (Chaerle et al., 2007; James and Sirault, 2012; Guretzki and Papenbrock, 2013). Furthermore, an influence due to pests and disease on the leaf temperature was observed (Allègre et al., 2007). Therefore, in this study, thermographic analysis was carried out to analyze early symptoms of stress caused by sulfur limitation or pathogen attack. The thermal imaging investigation was carried out with the camera T360 (FLIR Systems, Wilsonville, USA) according to Grant et al. (2006), in order to measure the surface temperature on plant leaves. For an optimal signal-to-noise ratio, the camera was turned on at least 30 min before the first thermographic picture was taken. For analyzing the pictures of three plants per cultivar and per treatment, the program ThermoCam Researcher 2.10 FLIR QuickReport 1.2 SP2 (FLIR Systems, Wilsonville, USA) was used. The parameters were set for each image to emissivity 0.95, reflected apparent temperature 22°C, atmospheric temperature 22°C, relative humidity 70% and distance 0.8 m.

### Chlorophyll fluorescence measurements

Chlorophyll fluorescence was determined by a PAM M series device and ImagingWin v2.32 software (Heinz Walz, Effeltrich, Germany). The measurements were performed with up to six areas of interest (AOI, points on the leaves where the measurement data points were taken) on different expanded leaves. Light curves using different photosynthetically active radiations (PAR) were examined as presented in the manufacturer's handbook. Because of the use of the filter plate IMAG-MAX/F, the effective PAR values were about 15% lower. Before taking the measurement, the plants were dark adapted for 20 min. The parameters F_v_/F_m_ (maximal PS II quantum yield) and Y(II) (effective PS II quantum yield) were analyzed (for background information: Baker, 2008; Sperdouli and Moustakas, 2012). F_v_/F_m_ values were obtained from the false-color images, created by ImagingWin software. Measurements (*n* = 5–8) were performed 1 h after light was switched on.

### Biomass measurements

For the analysis of the biomass, all plants of the cultivars were harvested, and material was divided into shoot, stem, and root categories, before being weighed. When weighing the roots, all soil particles were removed by washing the whole root system of one plant and drying it carefully with tissue. The plant material was dried in paper bags at 80°C for 4 day, and the dry mass (DM) was determined.

### Morphology

Ten sterilized seeds per plate were germinated for 5 day on three plates with Blake-Kalff medium, containing 1 mM MgSO_4_/0.010 mM sulfate. Additionally, on half of the plates 0.5 μl of a 1^*^10^6^ spore suspension was applied. The roots of the seedlings were sectioned in an investigation zone that lay 1–2 cm below the root crown. Pictures of the primary roots were taken with a camera installed on a binocular (Olympus SZ2-ILST, Tokyo, Japan). The root hair lengths of five plants were determined at the computer using a ruler.

### Metabolic analysis

#### Elemental analysis of plant material

For the analytical measurements, pooled samples were measured at least three times, up to six times. Dry plant material was ground to fine powder (MM 400, Retsch GmbH, Haan, Germany). About 38 mg of the ground powder was incinerated for a minimum of 8 h in a muffle furnace (M104, Thermo Fisher Scientific Corporation, Waltham, Massachusetts, USA) for each cultivar and treatment. After cooling the samples to room temperature (RT) (between 21 and 23°C), 1.5 ml of 66% nitric acid was added. After 10 min, 13.5 ml of ultrapure water was pipetted to the samples. The solutions were filtered (0.45 μm pore size, Carl Roth, Karlsruhe, Germany) and stored in vials at −20°C before final analysis. The samples were analyzed by inductively coupled plasma optical emission spectrometry (ICP-OES) (iCAP 6000 ICP Spectrometer, Thermo Fisher Scientific Corporation). Fluctuations of the results were around 5% for sulfur and phosphorus. Iron measurement results showed fluctuations of about 20%.

#### Sulfate determination and analysis of soluble thiol compounds

Sulfate was determined by capillary electrophoresis (CE) in the following way: 30 mg of deep-frozen, fine-ground plant material was solved in 700 μl HPLC grade H_2_O, mixed for 1 min, incubated at RT for 10 min, mixed again for 1 min and centrifuged for 10 min at 13,200 × g at 4°C. The supernatant was transferred to a new reaction tube, frozen overnight, and after thawing, centrifuged another 10 min. The supernatant was transferred to a 500 μl reaction tube and used for CE analysis (P/ACE™ MDQ Capillary Electrophoresis System with MDQ-PDA detector, Beckman Coulter, Krefeld, Germany). Separations were performed in a Beckman Coulter eCAP™ CE-MS capillary (fused silica, 75 μm i.d., 57 cm total length, 50 cm effective length). Before starting the analyses, the capillary was rinsed with HPLC grade H_2_O for 10 min and equilibrated with the background electrolyte Basic Anion Buffer for HPCE (Agilent Technologies, Waldbronn, Germany) at 14.5 psi for 10 min. Injection was done by applying 0.7 psi for 6 s. Separation of the samples was performed by applying 14 kV, 22°C, reverse polarity for 10 min. Samples were detected at 350 nm with a bandwidth of 20 nm. Calibration graphs for sulfate were generated with 78–10,000 μM Na_2_SO_4_. The detection limit for this method is about 10^−13^–10^−16^ mol. Evaluation of the electropherograms was done with Karat 32 7.0 software. The determination of thiols was done according to Riemenschneider et al. (2005).

#### Determination of phenols and flavonoids

To 50 mg of ground leaf material 800 μl of 80% methanol was added, mixed for 10 min and centrifuged for 5 min. The pellet was re-extracted three times with 400 μl methanol and the supernatants were combined. The whole sample was centrifuged and the supernatant stored at −70°C.

Based on the method of Dudonné et al. (2009), 100 μL of water was pipetted into a 96-well microtiter plate. Triplicates of 10 μL sample, blank (80% methanol) or gallic acid standard (5–250 μg mL^−1^) and finally 10 μL Folin Ciocalteu reagent were added. After incubation for 8 min and addition of 100 μL 7% sodium carbonate, the plate was incubated for 100 min and measured at 765 nm. Total phenols were calculated from a standard curve.

Flavonoids were analyzed based on Dewanto et al. (2002). To each well of a clear 96-well microtiter plate 150 μl water, 25 μl sample or catechin hydrate standard (10–400 μg ml^−1^) or 80% methanol as blank (in triplicate) and 10 μl 3.75% NaNO_3_ were added. After 6 min incubation, 15 μL 10% AlCl_3_ were added. After 5 min incubation, 50 μl 1 M NaOH was added and the total flavonoids were calculated from a standard curve based on the absorption at 510 nm.

#### Measurements of anthocyanins

For the determination of the anthocyanin content, 1 ml 1% HCl in methanol and 0.5 ml H_2_O were added to 50 mg of ground plant material and incubated overnight at 4°C. Then the samples were centrifuged for 15 min at 21,000 × g at RT. The supernatants were measured at 530 and 675 nm for the anthocyanin concentration and degraded products of the chlorophyll determination, respectively (Rabino and Mancinelli, 1986). The formula for the calculation was: c_anthocyanin_ = AU(A_530_-(0.25^*^ A_657_))/g FM.

### Sequence analysis

For the primer design, sequences homologous of *A. thaliana* DNA sequences for APR2 and APR3 sequences were searched in the *B. napus* database (Computational Biology and Functional Genomics Laboratory, 2014) using BLAST. The data bank uses parts of short homologous sequences (high-fidelity virtual transcripts; TC-sequences, tentative consensus sequences) to generate EST sequences (Quackenbush et al., 2000) that were used for the primer pair design (Primer Design version 2.2, Scientific & Educational Software, Cary, USA). For the design of the primer pairs for the amplification of cDNA fragments of sulfate transporter, the respective homologous sequences from *Brassica oleracea* were used (Buchner et al., 2004a), because the *B. napus* sequences were still not available. The primers were used to amplify cDNA fragments between 339 and 973 bp (Table 2).

**Table 2:** **Primers used in this study**.

**Primer pairs**	***B. napus* DFCI TC No**.	***A. thaliana* accession No**.	**Sequences from 5′ to 3′**	**bp**
P226BoST4;2s	–	At3g12520	CGTTCCATAAGTCACTCAGTC	968
P227BoST4;2as			GTGTACGCTTCTGGATACTGC	
P743_BnAPR2_for	TC162152	At1g62180	CAAGAAGGAAGATGACACCACC	377
P744_BnAPR2_rev			GCGAATCGACATCTCTATGCTC	
P745_BnAPR3_for	TC186950	At4g21990	CATCAAGGAGAACAGCAACGCA	339
P746_BnAPR3_rev			TCGGGAACACTAGTATCGTCGG	

*Known sequences from A. thaliana genes were used to find homologous gene sequences from B. napus or B. oleracea. Blasted sequences (compbio.dfci.harvard.edu) were used to create the primer pairs (Primer Design version 2.2). The annealing temperature was 55.3°C for all primer pairs. S, sense; as, antisense; for, forward; rev, reverse; DFCI, Dana-Faber Cancer Institute; TC, tentative consensus*.

### Northern blotting

Total RNA was extracted according to Sokolowsky et al. (1990) from ground plant material and quantified spectrophotometrically. Fifteen μg of the RNA were separated on 1% denaturing agarose-formaldehyde gels. Equal loading was controlled by staining the gels with ethidium bromide. After RNA transfer onto nylon membranes, they were probed with digoxigenin-labeled cDNA probes obtained by PCR (PCR DIG probe synthesis kit, Roche, Mannheim, Germany). To amplify the respective probes, the sequence-specific primers listed in Table 2 were used. The colorimetric detection method with nitroblue tetrazolium (NBT) and 5-bromo-4-chloro-3-indolyl-phosphate (BCIP) as substrates for alkaline phosphatase was applied. Quantitative analysis of the Northern blot results was done by GelAnalyzer^5^ (http://www.gelanalyzer.com). None of the common housekeeping genes was expressed under sulfur deficiency, pathogen attack, and diurnal rhythm in a constitutive way. Therefore, calculations were done in the following way: For each membrane, the band intensity of the first sample (0 h) was set to 100%. The intensities of the following bands were referred to the intensity of the first band.

### Statistical analysis

The biomass data were evaluated using a Three-Way ANOVA with DM as dependent variable (log-transformed to meet linear model assumptions such as normality and homogeneity of variances) and cultivar, infection, and S concentration as independent factors. Significance of factors and their interactions was assessed by means of *F*-tests; all interaction terms except cultivar: infection proved non-significant and were thus eliminated from the model. To pinpoint significant differences among factor levels, we applied Tukey tests (i.e., pairwise mean comparisons) controlling the rate of type I errors at 5%. In the presence of interactions, the Tukey comparisons were carried out separately for each level of the interacting factor.

A similar ANOVA model was fit to the (logarithmized) shoot-to-root ratios; here all interaction terms turned out to be non-significant, so we simplified the model to main effects only before performing Tukey comparisons.

Leaf temperatures and quantum yields were analyzed with ANOVA-type linear mixed-effects models including leaf-specific random effects to account for correlation among 10 replicated measurements from each of five leaves. These models could not be simplified due to all two- and three-way interactions of the factors cultivar, infection, and S concentration being significant and hence not omissible. In consequence, pairwise Tukey comparisons were carried out separately for each combination of factor levels.

All statistical computations were done in R 3.1.1 (R Core Team, 2014). The graphs were generated with SigmaPlot 12.5 (Systat Software, Inc., San Jose, CA).

## Results

### Morphology

**Figure 1:**
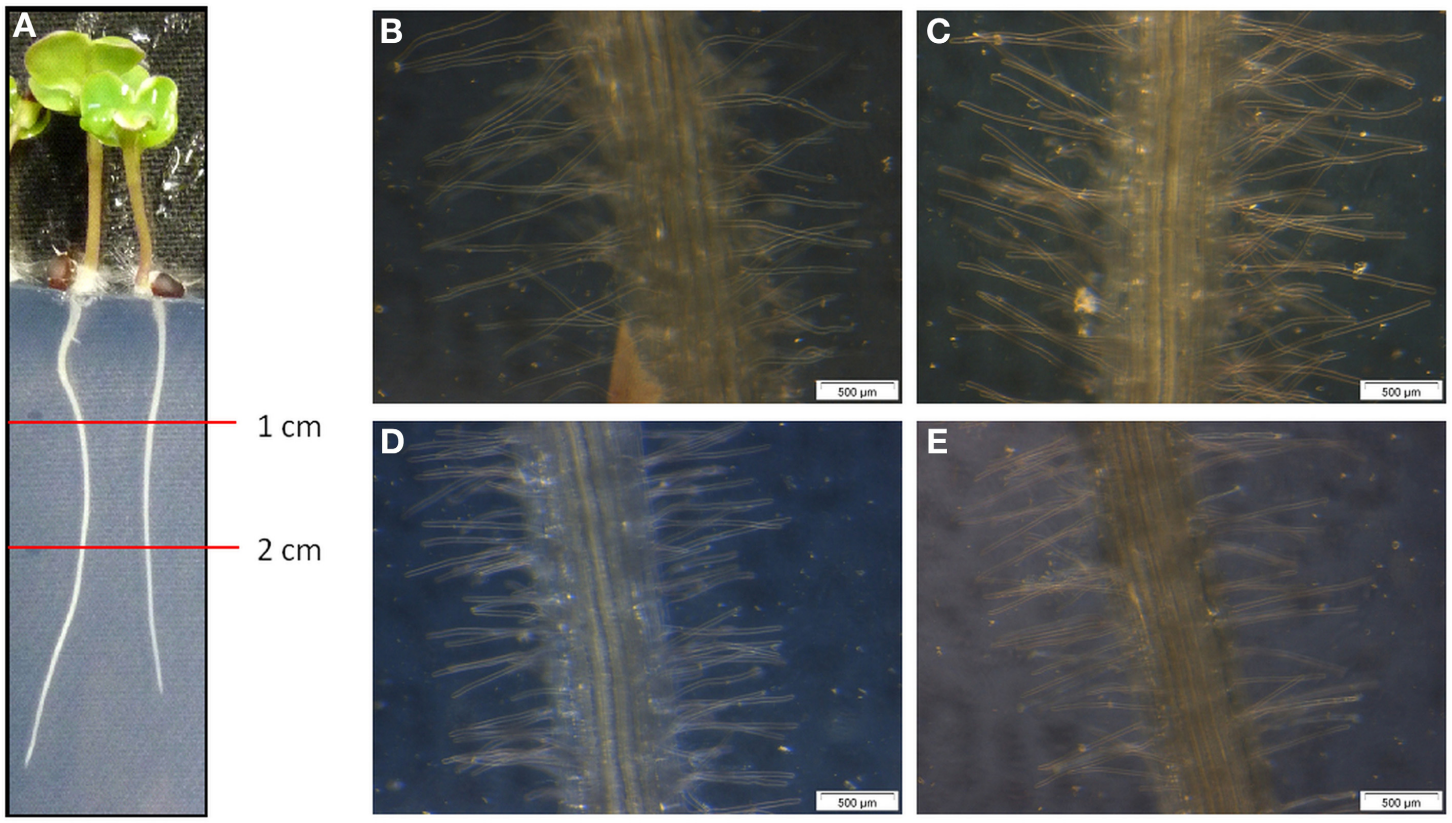
**Microscopy of root hairs**. At 5-day-old seedlings of winter oilseed rape the length of the root hairs was measured using a ruler and a binocular starting one cm below the root crown. **(A)** 5-day-old seedling, **(B)** Compass, **(C)** Exocet, **(D)** Genie, and **(E)** King10.

For the determination of the root morphology, sterilized oilseed rape seeds were grown for 5 day on Blake-Kalff medium with and without an adjusted spore suspension. The roots were separated in sections and the pictures were taken in the section one to two cm below the crown (Figure 1A). As visible on the pictures, Genie (D) has significantly shorter root hairs when comparing them with the cultivars Compass (B) and Exocet (C). The root hairs of Genie have an average length of 0.58 ± 0.08 mm. Figure 1 shows that the length of the hairs is different between King10 (E) and either of Exocet and Genie. As demonstrated by the statistical analyses (Table S1), there are no significant differences between the cultivars Compass and Exocet, with an average root hair length of 1.16 and 1.14 mm. No differences in the root length could be observed at seedlings grown with less sulfate or when incubated with *V. longisporum* spores in comparison to control conditions.

### Biomass production

**Figure 2:**
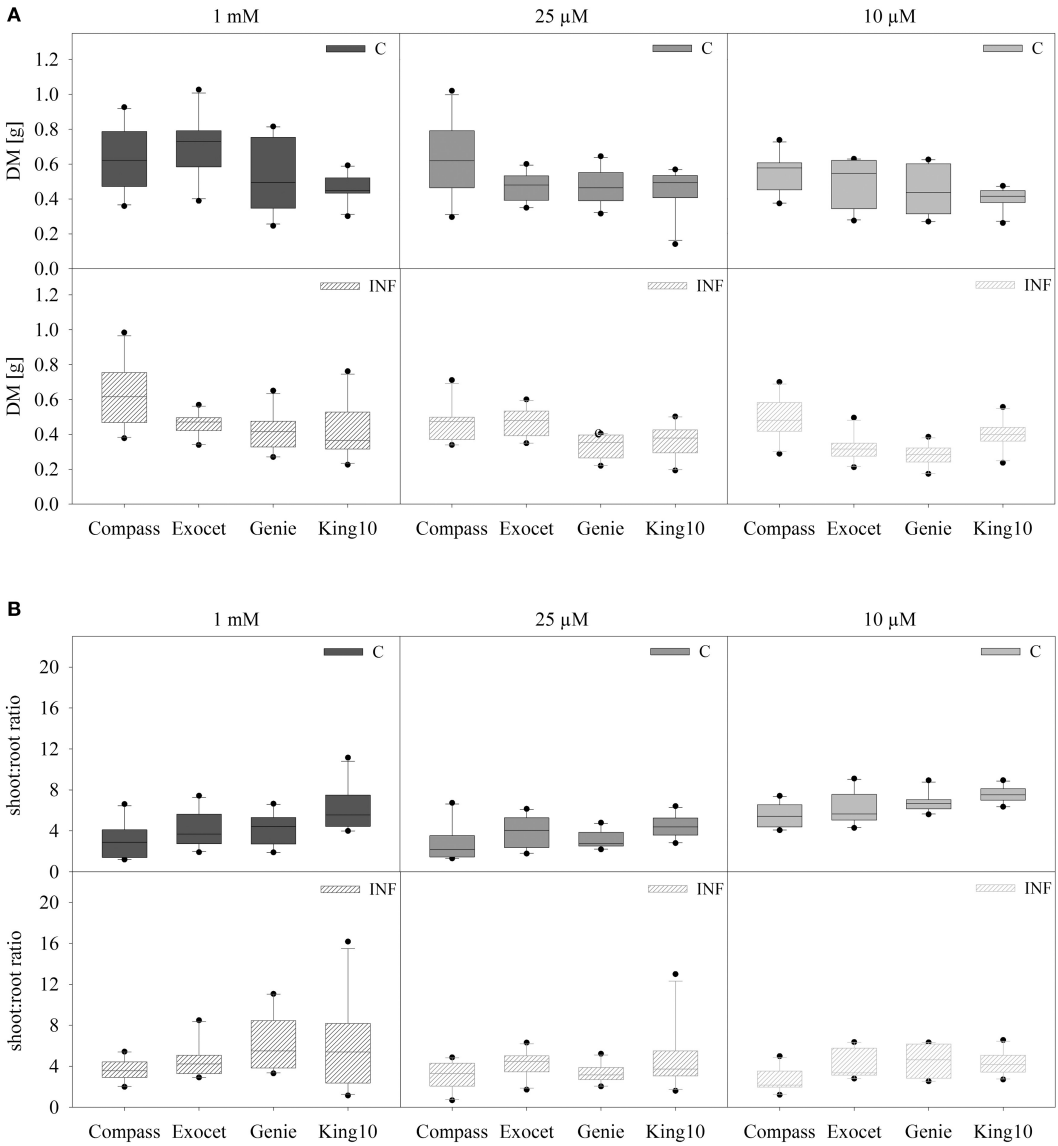
**Biomass analysis**. Seven-day-old seedlings of the cultivars were either mock-inoculated (C) or infected with *V. longisporum* spores (INF). Plants were grown under full sulfur supply (1 mM MgSO_4_) and under sulfur limitation (0.025 mM and 0.010 mM MgSO_4_) in a climate chamber for 14 dpi. The total dry mass (DM) **(A)** and the shoot to root ratio **(B)** of ten plants with five leaves fully expanded per treatment are presented. Data are represented as box plots. Filled boxes present non-infected plants (C) and striped boxes the corresponding results of infected plants (INF).

Because of the remarkable differences in the root hair length, the biomass of the organs and especially the root system was analyzed in more detail. Oilseed rape plants were cultivated as described, weighed and dried. No visible symptoms due to infection were observed. The total DM and the shoot to root ratio was calculated (Figure 2). The total DM of all non-infected (C) and infected (INF) cultivars is significantly decreased under sulfur deficiency, whereas no significant differences were observed between the deficiency conditions. Plants of the cultivar Exocet developed the highest leaf biomass with an average DM of up to 0.540 ± 0.147 g as mean values. Under sulfur limitation the biomass of leaves (−31.3%) and stems (−27.3%) decreased significantly in non-infected plants (data not shown).

Depending on the cultivar, the treatment with the fungus led to different DM. Highly significant reductions of DM in comparison to control were measured for Exocet and Genie. In infected plants, Exocet is the only cultivar showing significantly decreased biomass in all three organs (data not shown). Control plants of King10 developed significantly less DM compared to Exocet and Compass, Genie developed significantly less DM than Compass. Upon infection a significantly higher DM was determined for Compass in comparison to all other cultivars (Table S2).

As expected, the shoot to root ratio is significantly higher in plants grown under full sulfur supply compared to sulfur deficiency conditions, whereas the fungal infection has no effect. To distinguish Compass plants achieved the significantly lowest shoot to root ratio (Figure 2B, Table S3).

### Physiological measurements

**Figure 3:**
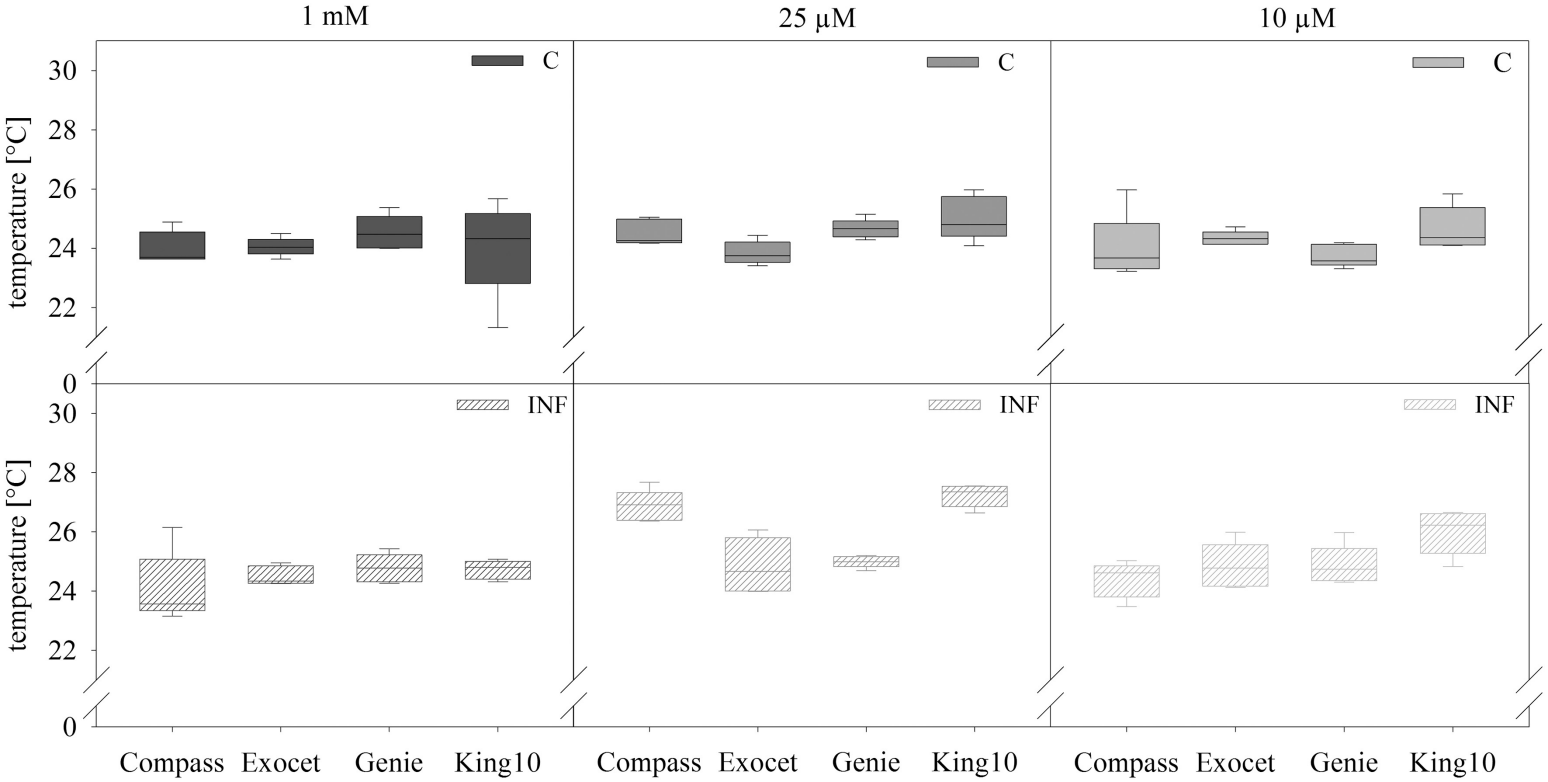
**Thermal imaging**. Pictures of plants with five leaves fully expanded treated as described for Figure 2 were obtained by thermography. Results show the temperature in °C of leaves and represent the data as box plots of five plants per treatment with 10 dots chosen on fully expanded leaves. Filled boxes present non-infected plants (C) and striped boxes the corresponding results of infected plants (INF).

To detect small effects of sulfur limitation and especially of pathogen attack on the cultivars at an early stage, physiological measurements were conducted. The non-invasive method of analyzing the leaf temperature with a thermo camera was used (Figure 3).

In non-infected plants, the fertilization of the different cultivars generated no significant differences in the leaf temperature. Only for plants grown under sulfur deficiency did the infection led to increasing leaf temperature. Statistical analyses revealed significantly substantial increased leaf temperature as compared with 1 and 0.01 mM MgSO_4_ in the cultivars Compass and King10 grown with 0.025 mM MgSO4 after 14 dpi. The temperature increased significantly in Compass and King10 (0.025 mM MgSO_4_), whereas the temperature decreased under 0.01 mM MgSO_4_ in Compass in comparison to the other cultivars. Thus, differences were measured with low sulfur supply (0.01 mM) and infection, in which King10 showed the highest and Compass the lowest temperature. Results obtained from infected plants with the thermo camera clearly indicate that significant differences between the different cultivars only occur under sulfur limitation (Table S4).

**Figure 4:**
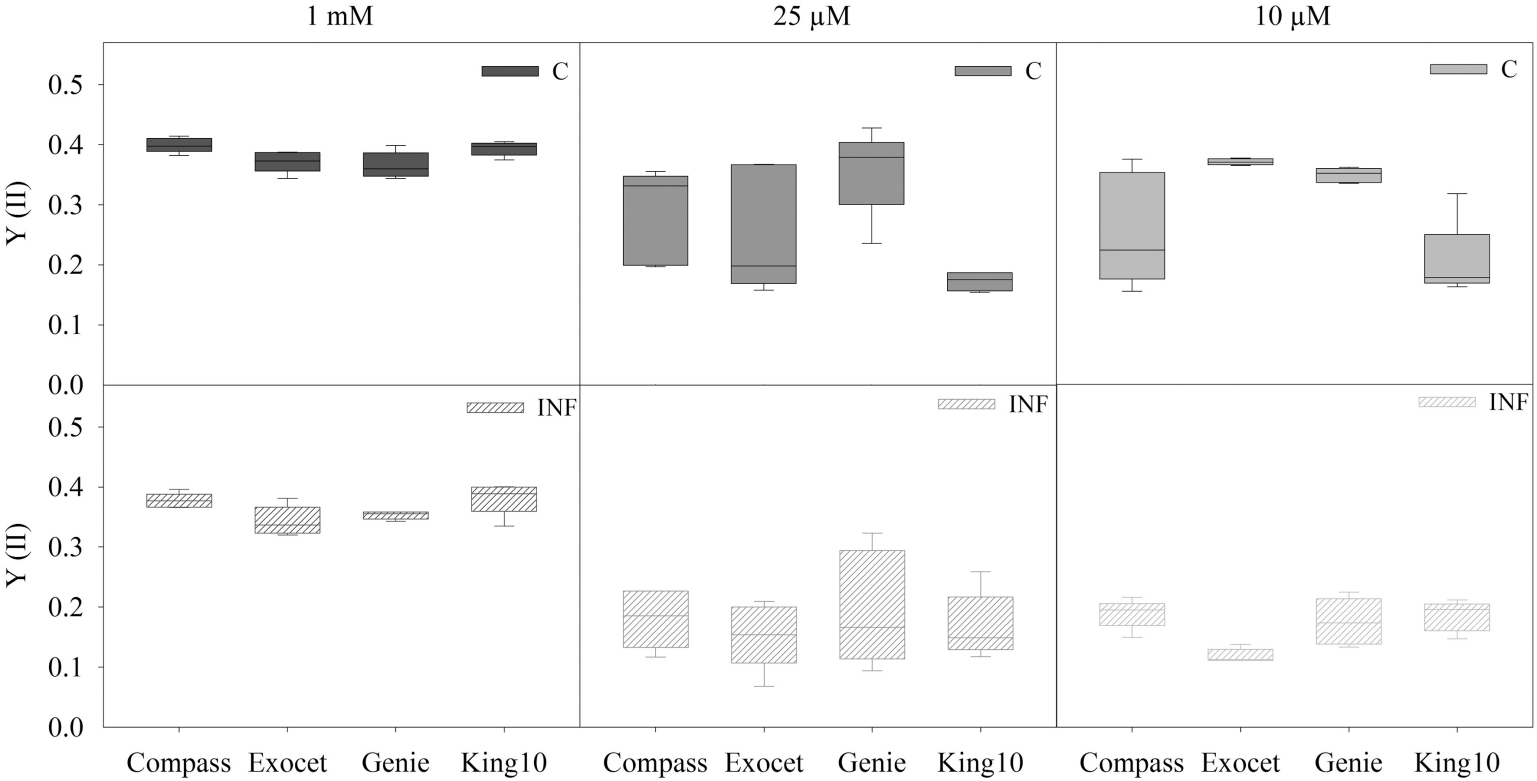
**Chlorophyll fluorescence**. The chlorophyll fluorescence measurements were done by PAM imaging. Five plants (five leaves fully expanded) per sulfate application and pathogen treatment (described for Figure 2) were determined by calculating the yield (YII) out of two areas of interest (AOI). The performances were obtained for 10 min after 20 min of dark adaptation. Data are represented as box plots. Filled boxes present non-infected plants (C) and striped boxes the corresponding results of infected plants (INF).

Chlorophyll fluorescence measurements were done with non-infected and infected plants grown with different sulfur concentrations (Figure 4). Independently of the sulfur supply, the F_v_/F_m_ values range between 0.714 and 0.831 (data not shown), indicating photosynthetic activity in the same range when grown at higher or lower sulfur concentrations (Kitajima and Butler, 1975). Under full sulfur supply, Compass and King10 performed better (around 5%) than Exocet and Genie. A slight decrease of the quantum yield was observed in Compass and Exocet under sulfur limitation. The sulfur limitation decreased the quantum yield of photosystem II in King10 strongly. The quantum yield of Genie plants remains almost constant.

No significant differences were obtained for plants grown under full sulfur supply after infection. Infection with the fungus *V. longisporum* led to a significant decrease of the quantum yield of plants grown under sulfur limiting conditions from approximately 4.5–41%. The performance of King10 was not significantly influenced by infection (Table S5).

### Metabolic analysis

#### Elements

**Figure 5:**
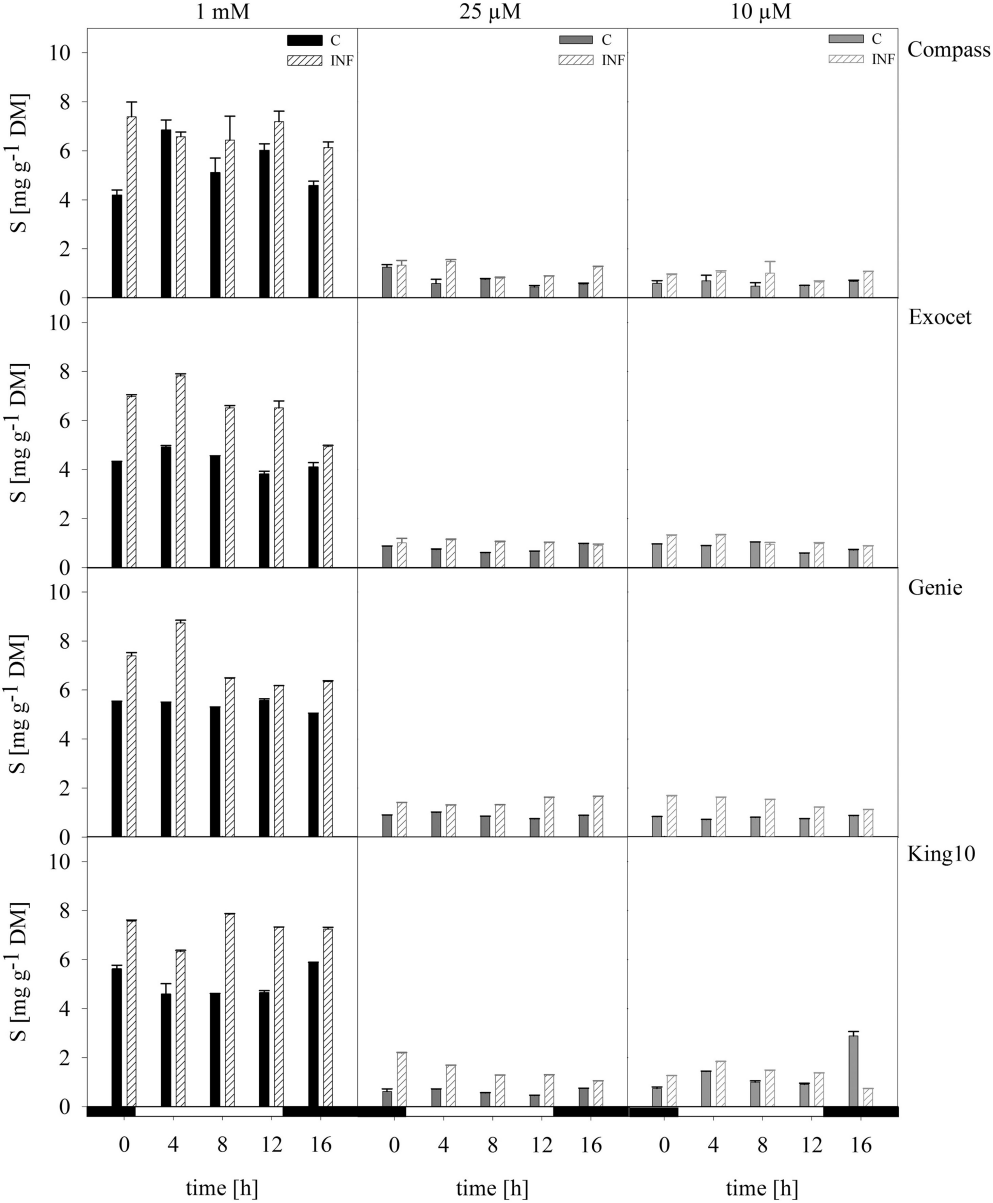
**Elemental sulfur**. The elemental sulfur was measured in dried plant material by ICP-OES. Seven-day-old seedlings of the cultivars were mock-inoculated or infected with *V. longisporum* spores. Plants were grown under full sulfur supply (1 mM MgSO_4_) and under sulfur limitation (0.025 mM and 0.010 mM MgSO_4_) in a climate chamber for 14 dpi. Three plants with five leaves fully expanded per treatment were harvested in a diurnal rhythm every 4 h starting 1 h before light was switched on (0–16 h). For measuring the material was dried. Data calculated as mg g^−1^ DM represent the mean of three dependent technical replicates ± SD. Filled bars represent the mock-inoculated plants (C) grown with 1 mM; 0.025 or 0.01 mM MgSO_4_ and the striped bars show the corresponding results of infected plants (INF).

Plant material of three plants per time point were dried and used for the elemental analysis (Figure 5). The control plants fed with 1 mM sulfate showed amounts in a range between 4 and 7 mg sulfur per g DM (Figure 5). Under sulfur limitation in all cultivars, the sulfur content decreased below 2.18 mg g^−1^ DM. Calculated over the time range of the day Genie incorporated the highest amounts of sulfur in control plants followed by Compass. On average, Exocet accumulated 20% less sulfur than Genie. King10 incorporated 3.78 times less sulfur in plants with 0.01 mM than control plants. In control plants of Compass sulfur, values show deviations over the day with a maximum at 4 h with 7.20 mg g^−1^ DM.

Infected plants of all cultivars fed with 1 mM MgSO_4_ incorporated more sulfur in the shoots than the control plants. Interestingly, King10 incorporated over the whole time range higher sulfur amounts (7.26 mg sulfur g^−1^ DM) in the plants grown with full sulfur supply. Under sulfur limiting conditions plants infected with the fungus incorporated more sulfur than the control plants. In Genie, the sulfur content increased by 30, 66, and 80% compared with the control plants fed with 1, 0.025 and 0.01 mM MgSO_4_.

Subsequently, iron and phosphorus were analyzed because both elements are, e.g., indispensable for energy transfer and structural components (Expert et al., 2012). Deficiencies of phosphorus are common and frequently limit canola yields (Prabhu et al., 2007).

The plants of the cultivars incorporated under control conditions approximately 0.03–0.11 mg iron g^−1^ DM (Figure S1). Apart from a few sample values, the iron content decreased in plants fed with less sulfur except in the cultivar Compass. The highest amounts of iron were measured in the plants fed with 1 mM sulfate in King10 (0.06–0.11 mg iron g^−1^ DM). The iron content increased by 16.5–63%, due to infections in all cultivars.

In the cultivars, only slight differences were observed in the phosphorus content (Figure S1). Exocet showed the highest phosphorus values without big fluctuations. In Genie plants, the phosphorus in mg g^−1^ DM increased in the plants fed with 0.025 mM SO^2−^_4_. The values in the line King10 remained without high fluctuation; the content of phosphorus increased under the condition of sulfur limitation in non-infected plants.

As a tendency, Compass showed less incorporated phosphorus compared to the other cultivars. The measurements furthermore showed hints that the phosphorus content in infected plants increased.

#### Sulfate

**Figure 6:**
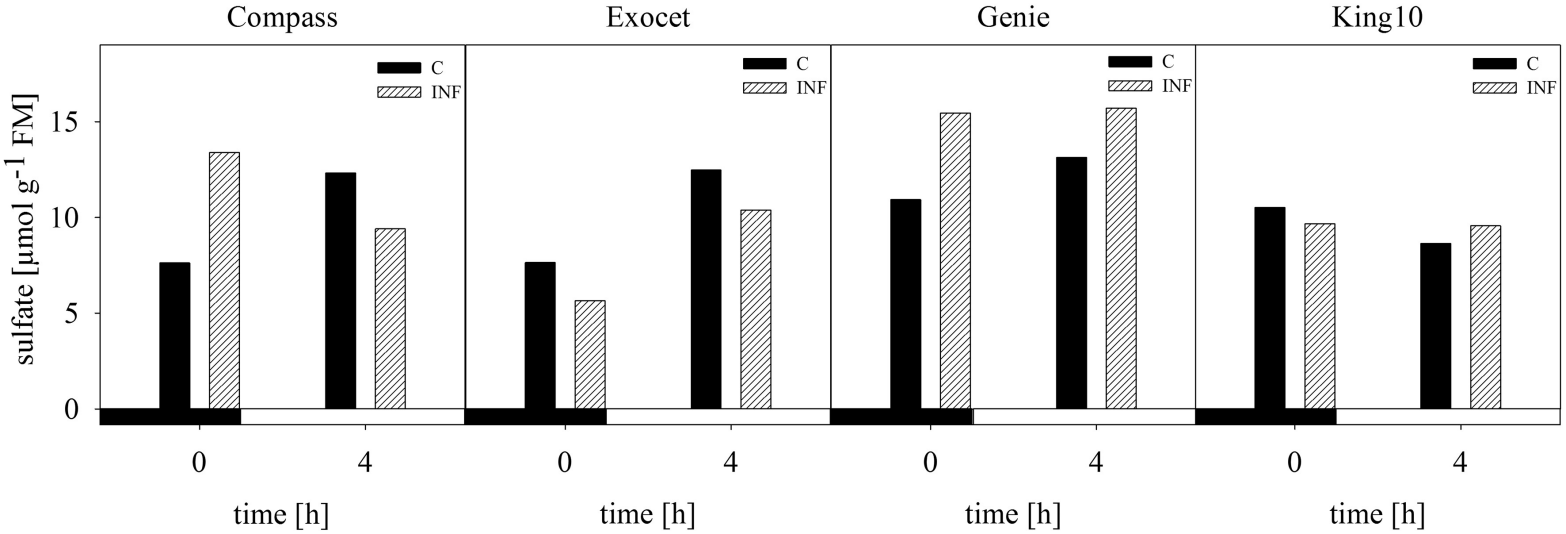
**Determination of sulfate**. Sulfate content was measured by using capillary electrophoresis. Leaf material from non-infected and infected plants with five leaves fully expanded grown with 1 mM MgSO_4_ and collected at 0 and 4 h was used. Sulfate amount was calculated in μmol g^−1^ FM and data represent the result of one measurement. Filled bars represent the control plants (C) and striped bars indicate infected plants (INF).

The anion sulfate which is taken up by the roots was measured in leaves from non-infected and infected plants using capillary electrophoresis. In the samples from plants grown with 0.025 and 0.01 mM MgSO_4_, the sulfate contents were below the detection limit of the method used. Exemplary for dark and light conditions, the amount of sulfate, given in μmol g^−1^ FM, was analyzed at 0 (dark) and 4 h (light) in control and infected plants (Figure 6). For non-infected plants of the three varieties, an increase in the sulfate amount from the harvesting time point in the dark to the harvesting time point in the light was measured, whereas in line King10 the sulfate content slightly decreased. At 0 and 4 h, Genie showed the highest values with 10.93 and 13.14 μmol g^−1^ FM, respectively. An increase in the sulfate content in infected Compass and Genie plants at 0 h up to 43 and 30% was observed. In contrast, Exocet and King10 did not show any increase of sulfate in infected plant at 0 h. In Genie, the highest amounts of sulfate (about 16 μmol g^−1^ FM) were observed after infection, both in light and dark.

#### Thiols

**Figure 7:**
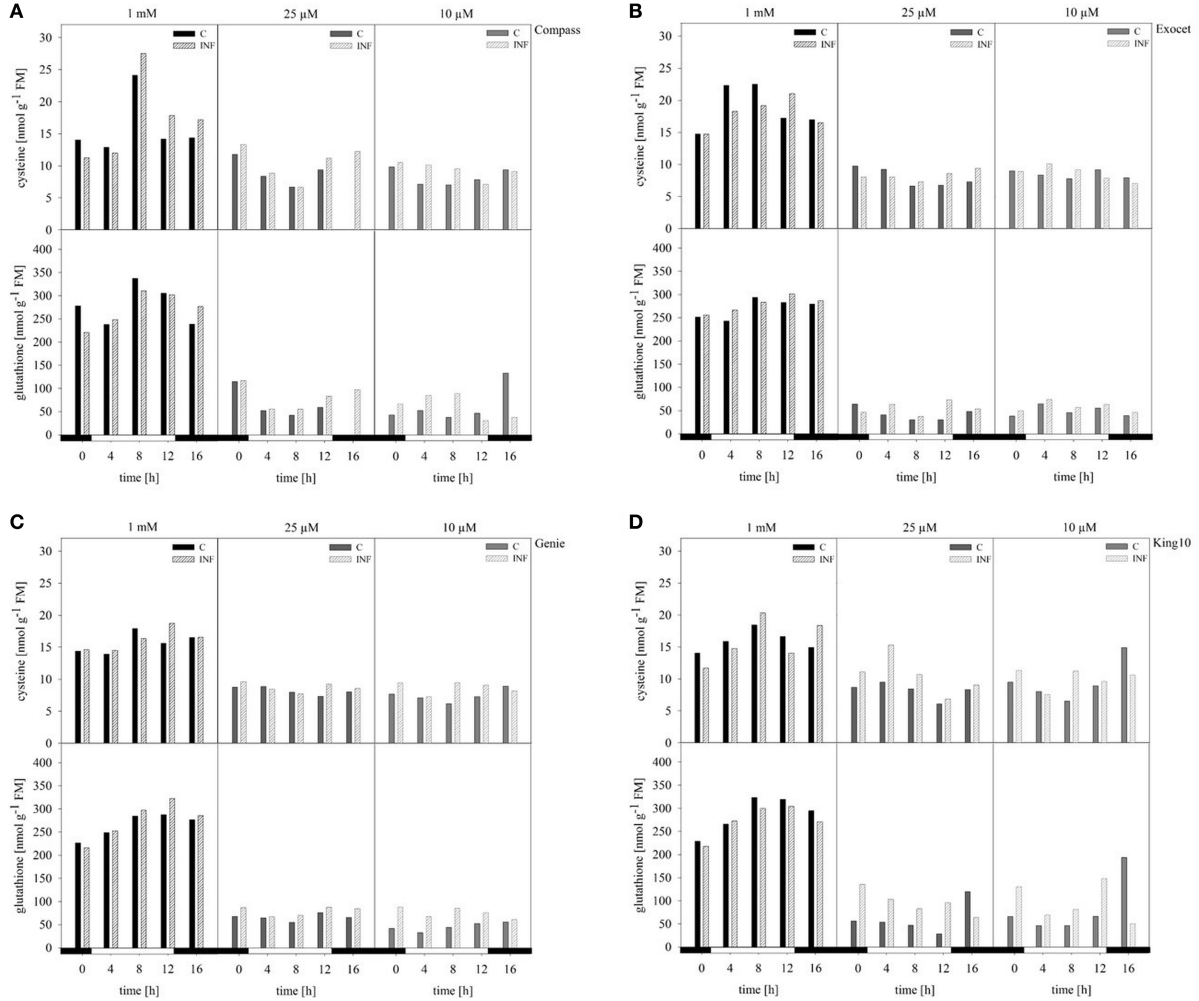
**Thiol contents**. The cysteine and glutathione contents were determined in plants (five leaves fully expanded) treated and collected as described for Figure 5 by HPLC. The thiol contents were calculated in nmol g^−1^ FM in the cultivars **(A)** Compass, **(B)** Exocet, **(C)** Genie, and **(D)** King10. Filled bars represent the control plants (C) for different sulfur fertilization and striped bars indicate infected plants (INF).

The compounds cysteine and glutathione, which contain reduced sulfur, were analyzed in control and infected plants by HPLC. Under full sulfur, supply an increase in the cysteine content was measured ranging from 11.25 up to 27.52 nmol g^−1^ FM over the day. Highest amounts were observed after 8 h, and lowest amounts at 0 and 16 h (Figure 7). When comparing the amounts of cysteine among the cultivars, Exocet accumulated with full sulfur supply the highest amounts of cysteine. In plants grown under sulfur limitation, the cysteine content decreased by more than 30%. The lowest levels were obtained in the middle of the light phase. The glutathione content showed similar fluctuations over the day. However, the decrease under sulfur limitation by up to 90% is more drastic.

In infected plants grown under full sulfur supply, the amounts of cysteine and glutathione increased only slightly. Under sulfur deficiency, more cysteine and glutathione is accumulated in the leaves of infected plants. Among the cultivars, King10 showed the highest amounts of accumulated thiols in leaves of infected plants. After 8 h, the plants of King10 accumulated up to 40% more cysteine than the control plants under sulfur deficiency. Infected plants of King10 accumulated three times more glutathione than control plants.

#### Phenols and flavonoids

**Figure 8:**
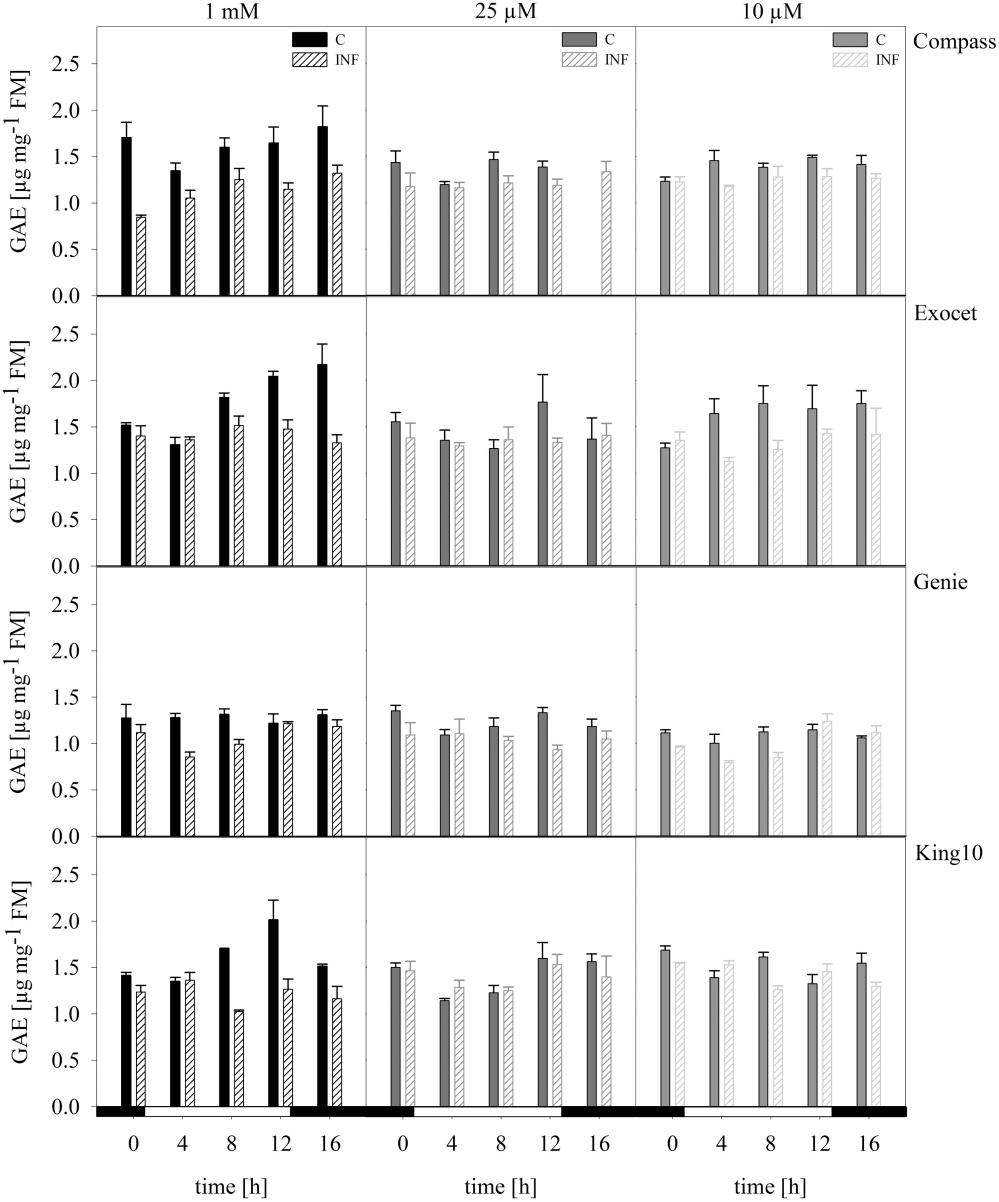
**Total phenols**. The total phenol content was photometrically measured in plants with five leaves fully expanded of all cultivars. Plants for the measurement were treated and collected as described for Figure 5. The results are shown in μg g^.-1^ FM as mean of three technical replicates ± SD. For quantification gallic acid was used as a standard. Filled bars represent the control plants (C) for different sulfur fertilization and striped bars indicate infected plants (INF). GAE, gallic acid equivalent.

***Total phenols*.** Phenolic compounds are bioactive components and are discussed to have high health-promoting activity. The content and composition of phenolic compounds can be used to distinguish among plant cultivars and varieties (Klepacka et al., 2011). Figure 8 shows the phenolic contents of the cultivars. The results of the control plants showed that they accumulated the highest amounts of phenolic acids. Only slightly decreasing amounts were observed in plants grown under sulfur deficiency. The control plants of the cultivar Genie showed thereby the lowest levels (Figure 8). In infected plants, the phenolic levels were reduced independently of the sulfur supply.

***Total flavonoids*.** Measurements of the total flavonoid content (Figure S2) showed that there were only slight differences. The values ranged between 0.6 and 1.4 μg catechin equivalent per mg FW. Interestingly, in the plants of Genie, the control plants showed more accumulated flavonoids than infected plants, independently from the amounts of sulfur applied. By contrast, King10 showed the lowest flavonoid levels in the control plants and under sulfur limitation (0.025 mM MgSO_4_), the infected plants produced more flavonoids than the control plants.

**Figure 9:**
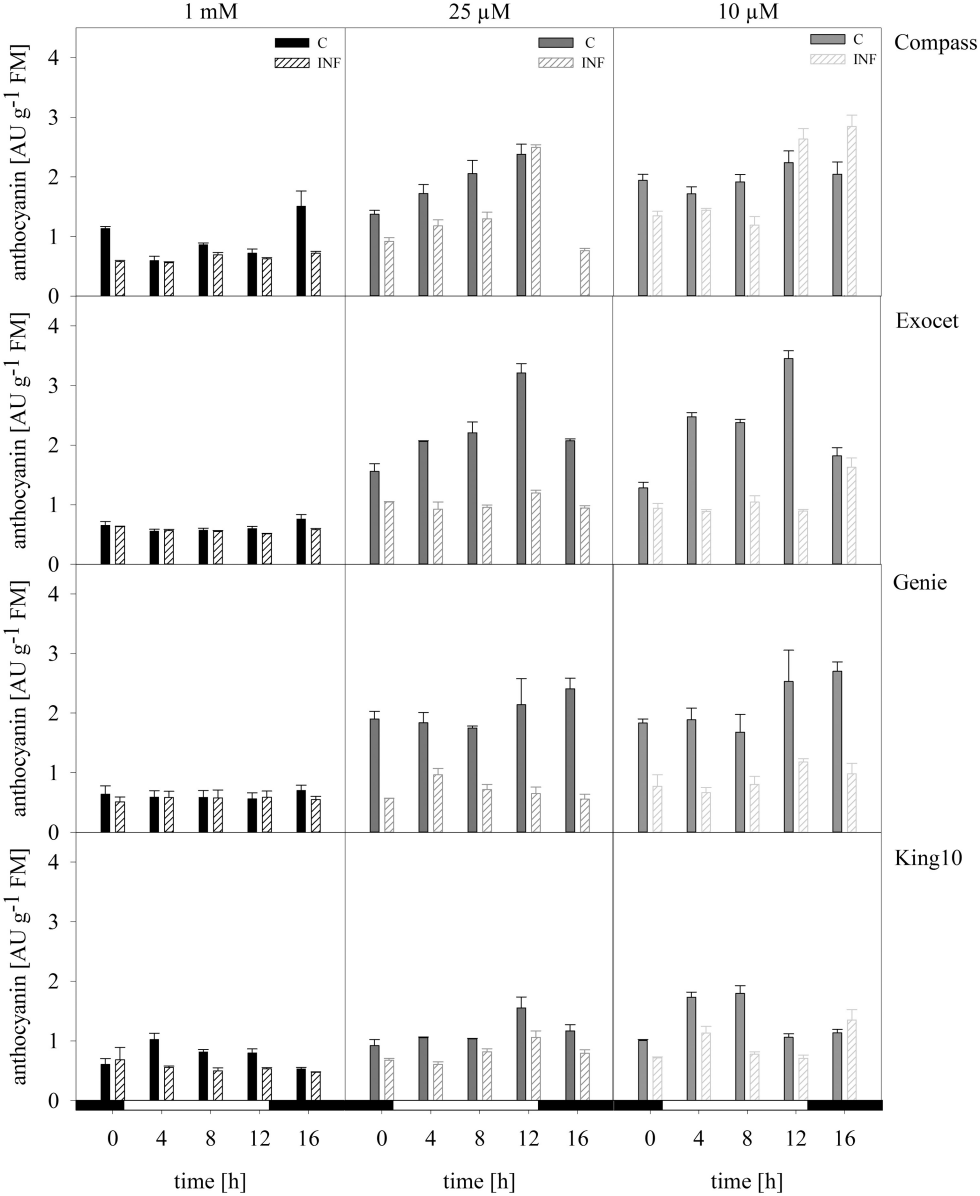
**Anthocyanins**. The anthocyanins were extracted from the fresh material of plants with five leaves fully expanded treated as described for Figure 5. The content was calculated in AU g^−1^ FM representing the mean of technical replicates, which were measured three times. Filled bars represent the control plants (C) for different sulfur fertilization and striped bars indicate infected plants (INF). AU, absorbance units.

***Anthocyanins*.** Anthocyanins are a subgroup of the flavonoids. The values range between 0.56 and 3.45 absorption units (AU) of anthocyanins per g FM for the control plants (Figure 9). The lowest levels were determined in the control plants (0.56–1.37 AU g^−1^ FM). Plants grown under sulfur limitation showed increased levels. Notably, Exocet reached the highest values.

Infected plants especially from Exocet and Genie showed no differences to control plants at full sulfur supply. The anthocyanin levels increased only slightly under sulfur limitation. With less sulfur, the anthocyanin levels in King10 and Compass increased, but only in Compass the amounts exceed the levels of the non-infected plants at time points 12 and 16 h.

### Northern blot analysis

**Figure 10:**
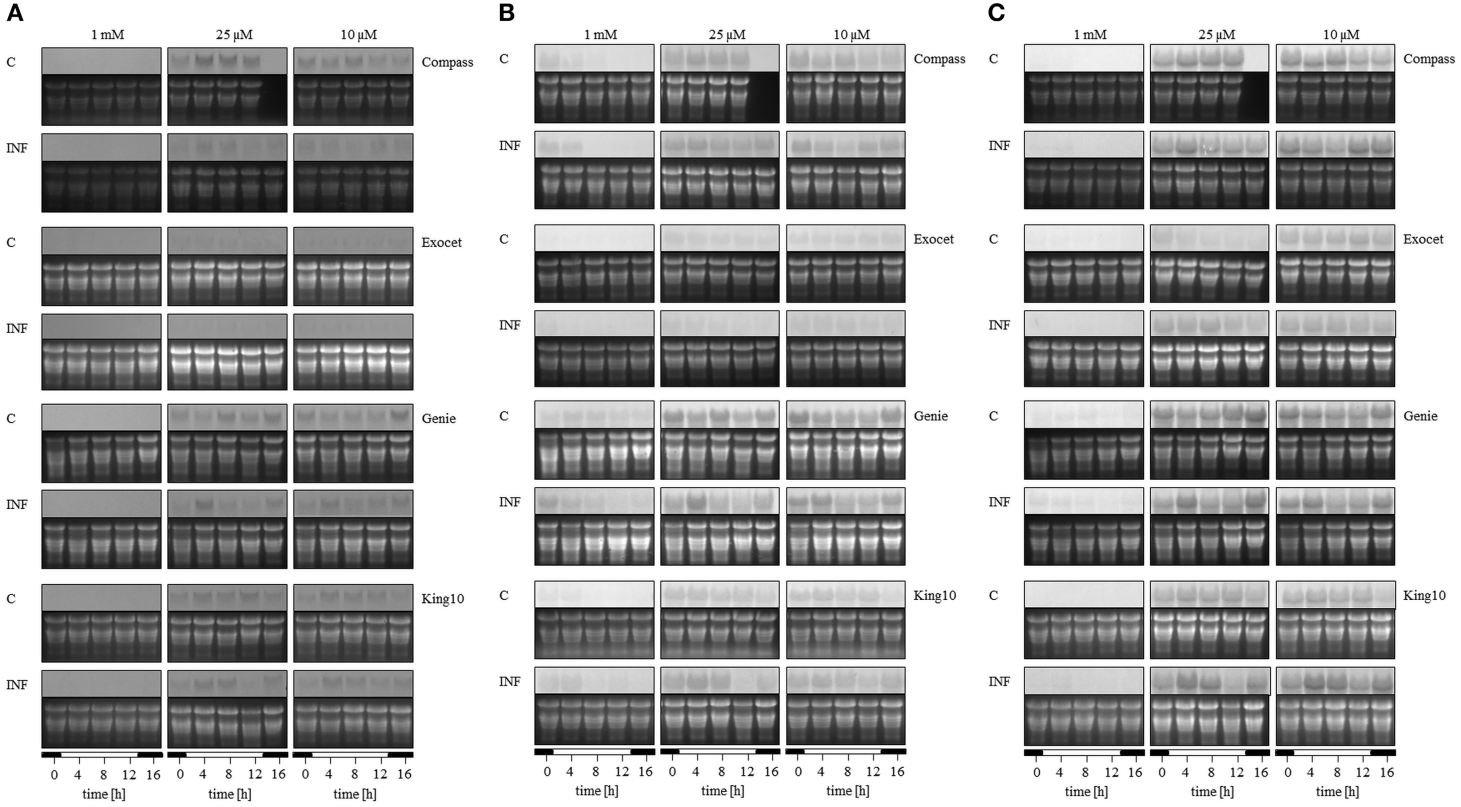
**Expression analysis**. The expression of **(A)** *SULTR4;2*, **(B)** *APR2*, and **(C)** *APR3* was analyzed by Northern blotting in plants (five leaves fully expanded) treated and collected as described for Figure 5. Total RNA were isolated and transferred onto membranes. The detection of mRNA was done with probes labeled with digoxigenin. Abbreviations for probes: *APR*, adenosine phosphosulfate reductase; *SULTR*, sulfate transporter.

To analyze the key steps of sulfur assimilation in the oilseed rape cultivars, the expressions of *sulfate transporter 4;2* (*SULTR4;2*), and two *APR* genes were determined (Figures 10 A–C, Figure S3). As an indicator for sulfur-induced stress the expression of *SULTR4;2* was analyzed (Parmar et al., 2007). In addition, this is the only transporter expressed in leaves that rapidly responds to S deficiency (Buchner et al., 2004b). Only under sulfur limitation did the expression of the tonoplast-localized *SULTR4;2* increase strongly, indicating that S fertilization in our experiments is sufficient. The highest degree of up-regulation was detected in Compass. Light seemed to influence the expression slightly in the time range 3–7 h after light was switched on. Described as one of the primary regulatory points in sulfate reduction, adenosine 5′-phosphosulfate (APS) is reduced to sulfite catalyzed by APS reductase. Therefore, the homologs of both *APR2* and *APR3* were analyzed because both isoforms reveal a different expression behavior in *A. thaliana* (Kopriva et al., 1999). In *B. napus*, the expression of both genes was influenced by the sulfur status of the plants. Under limitation, the *APR* expression increased in all cultivars. The lowest degree of up-regulation was detected for *APR2* in Compass, the most in Genie. Regarding the expression of *APR3*, the highest increase under sulfur limitation was determined in King10.

In the experiments, the expression of *BnSULTR4;2* is affected by the pathogen. Especially under sulfur limiting conditions, the expression decreased, most strongly in Compass. The pathogen controls the expression of *APR2* in the following intensity order: Compass, Exocet and Genie. The line King10 showed higher expression levels of *APR2* than Genie, but here the expression is clearly up-regulated in infected plants. In the case of the expression pattern of *APR3*, in Genie and King10 the highest expressions in infected plants were detected 3 h after light was switched on. The difference among the *APR3* expression in infected and non-infected plant was higher in King10 than in all other cultivars.

### Analysis of the amplified DNA fragments of *B. napus* genes

The analysis of the four different *B. napus* cultivars revealed a number of different reactions with respect to the sulfur supply. Therefore, we investigated whether the cultivars differ in the sequences of key genes in sulfur metabolism. The respective DNA fragments of about 500 bp were amplified by PCR from the transcribed cultivar-specific cDNAs, cloned and sequenced. A considerable difference among *APR2* and *APR3* sequences was observed (78%), indicating the correct choice of primer pairs for amplification of specific fragments. In comparison to the *B. rapa* consensus, sequences the identity was for APR2 93% and APR3 96%, respectively. In comparison to *B. oleracea*, the identity was 100% for both, APR2 and APR3. The alignment of the *APR2* and *APR3* fragments from the four cultivars revealed no different bases among them. A longer fragment was amplified from the *B. napus* sulfur transporter *SULTR4;2*. The identity of the 975 bp fragment chosen was 99% between the sequences from Genie and King10. The identity of these *SULTR4;2* fragments with the homologous partial sequence of the *B. oleracea* transporter was 97% for both sequences.

## Discussion

### Which cultivars show the highest resistance against pathogen infection?

Before we can decide which cultivar is the most promising according to our results, we need to discuss first the recorded data for biomass, chlorophyll fluorescence and leaf temperature. Therefore, the best cultivar in this study is characterized by high biomass of all organs at all conditions chosen, both sulfur limitation and infection by *V. longisporum*, indicating general plant health. High biomass is accompanied by comparatively low leaf temperature indicating intact stomatal closure reaction and an intact water status. The photosynthetic parameters determined should be close to the range of the controls revealing intactness of the photosynthetic apparatus. Unfortunately, quantification of the infection rate is currently not possible because fungal DNA concentrations seem to be below the detection limit, as was also reported by Enyck et al. (2009). However, biometrical parameters such as biomass reduction and PAM data clearly indicate successful infection and spreading of the fungus in the infected plants.

Compass and Exocet have the largest root system (Table 1). Our results show that Compass and Exocet have significantly longer root hairs (Figure 1). Schröder (2013) advised that high-performing plants root very deeply with a production of up to 5 mm long root hairs. The root hairs are essential for a better uptake of nutrients. In greenhouse experiments, a sufficient sulfur supply was added to an intact root system, resulted in a reduced fading away of roots and led to an increased efficiency of nutrient and water use (Schröder, 2013; Grierson et al., 2014). The biomass measurements in this study are in agreement with these observations (Figure 2). With full sulfur supply, cultivars Compass and Exocet showed the highest leaf biomass. Under sulfur limitation, non-infected plants of both cultivars showed also the highest leaf biomass (data not shown). However, for all cultivars a significant reduction of DM and shoot to root ratio was obtained under sulfur limiting conditions. Interestingly, in previous experiments, it was shown that the fungus had no influence on the shoot to root ratio but only on the total DM of the plants after 14 dpi as described in Enyck et al. (2009). In experiments done with *B. napus* and *V. longisporum*, the fungus did not overcome the hypocotyl barrier until 21 dpi, although the plants showed massive stunting of the stem and mild leaf chlorosis. A significant decrease of stem biomass was observed after 28 dpi (Floerl et al., 2008). In experiments done with more than 30 high-performance oilseed cultivars, it also was shown that *V. longisporum* influenced the plant growth. Infected cultivars showed reduced plant growth compared with control plants (Burlacu et al., 2012). However, in experiments we performed with plants grown for 21 day under sulfur deficiency and infection (21 dpi) showed very strong stress symptoms; therefore we disregarded these measurements and decided to analyze under the conditions described in this study. According to our results, Exocet seems to be more susceptible to fungal infections, whereas the biomass of Compass suggests to be the best performing cultivar.

Baker and Rosenqvist (2004) concluded that the reduction of sulfur levels in sugar beet had to reach sulfur starvation level before any changes were detectable in chlorophyll fluorescence parameters, e.g., in F_v_/F_m_. That leads us to the conclusion that our plants were grown only under sulfur limitation, and not sulfur starvation, and the influence on the maximum quantum yield remained low. Interestingly, the photosynthetic performance of King10 is significantly reduced under sulfur limitation. In contrast, the general fitness of Genie was influenced less by sulfur limitation as demonstrated by steady activities of the photosystems (Figure 4). However, after fungal infection the quantum yield in photosystem II was significantly reduced under sulfur limitation in all cultivars. It was shown before that the quantum yield of the photosystems decreased in plants infected with *V. longisporum*, but neither the nitrogen nor the sulfur or phosphorus amounts accumulated differently in non-infected and infected plants (Floerl et al., 2008). In conclusion, the fungus *V. longisporum* did not influence the photosynthetic activity of the plants under full sulfur supply. These results confirm the principle idea of SED (Rausch and Wachter, 2005). The sulfur status did not influence the leaf temperature significantly. Compass, Exocet and Genie, have a lower leaf temperature than the control plants when additionally infected with *V. longisporum*. At decreased applied sulfur amounts, the temperature was slightly higher, at least at 0.025 mM MgSO_4_. In experiments performed by Muneer et al. (2014) with 8-week-old *B. napus* plants grown under different sulfur supply for 5 or 10 day, the stomata were closed under sulfur limitation. Photosynthesis rates and stomatal conductance were decreased. Previously, it was observed that plants suffering from (abiotic) stress have a higher leaf temperature than non-stressed control plants (Guretzki and Papenbrock, 2013). Obviously, the thermographic analysis reveals different results, depending on stress and environmental conditions, but often leads to a clear differentiation between control and treatment.

### What is the role of sulfur, sulfate and sulfur-containing compounds in *Verticillium* defense?

Interestingly, the sulfur content itself was generally higher in infected plants than in non-infected plants, independent on the cultivar, indicating a specific increase of sulfur uptake and accumulation induced by pathogen attack. Looking at the accumulated total sulfur, line King10 incorporated the highest amounts of sulfur, in particular, after infection and under low sulfur application (Figure 5). Although the variety Genie performed worst compared to the other cultivars, it incorporated high amounts of sulfur, especially in non-infected plants, and in the same range as King10. All cultivars showed fluctuations of the sulfur content, especially Compass with full sulfur supply. Results from Huseby et al. (2013) showed a diurnal regulation of the sulfate uptake and reduction which corresponds with our results. Especially, the large increase in total sulfur contents at the beginning of the light phase is remarkable. The contents were analyzed several times in various experimental set ups and also by other experts (data not shown), resulting in consistent results. One could speculate that sulfate is taken up at the beginning of the light phase, reduced, and then later superfluously reduced sulfur is released as volatile compounds, such as sulfide, via the leaves and also via the soil. However, these assumptions need further investigation. An accumulation of elemental sulfur in the xylem vessels of tomato plants infected by *V. dahliae* was reported by Williams et al. (2002). Our results indicate an accumulation of total sulfur in *B. napus* leaves after infection with *V. longisporum*. Are these accumulations of total sulfur an indication for comparable defense strategies in both plant-pathogen systems? These results further support the hypothesis of SED.

Results from Huseby et al. (2013) showed a diurnal regulation of the sulfate uptake and reduction which corresponds with our results. In our results, the sulfate content was higher in light than in the darkness. The factor light outweighs the factor infection (Figure 6). In parallel, the expression of the sulfate transporter *BnSultr4;2* was increased during sulfur-limiting conditions with a maximum degree of expression during the light period (Figure 10A). It is clear that sulfate was taken up during periods of active photosynthesis. It was shown previously in experiments done with 4-weeks-old *B. napus* plants, that due to the sulfur limitation, the sulfate concentrations decreased at variable rates, at first in roots and young leaves, then in the middle leaves, and later in the oldest leaves. In parallel the sulfate transporter *BnSultr4;2* was first expressed in roots and in young leaves (Parmar et al., 2007). Interestingly, in our system, the expression of the sulfate transporter *BnSultr4;2* was influenced by the pathogen. However, in contrast to our expectations in all cultivars the mRNA levels of *BnSultr4;2* were lower in infected plants than in non-infected plants. In the context of SED higher mRNA levels of *BnSultr4;2* were expected due to a higher demand for sulfate and subsequent biosynthesis of sulfur-containing defense compounds. One could postulate that *V. longisporum* directly influences the plant's gene expression to prevent availability of sulfate, a prerequisite for the induction of SED. That needs to be investigated in future experiments.

Even though we do not know the full sequence of the isoforms of *APR* in *B. napus*, our results implied that the mRNA levels of *BnAPR2* are more strongly regulated by light than the levels of *BnAPR3*, especially visible in Genie under sulfur-limiting conditions. These results are in agreement with results obtained by Kopriva et al. (1999) and Huseby et al. (2013): Both the expression of three *APR* isoforms and of APR enzyme activity are diurnally regulated and by sulfate availability. In our experiments, the expression of both *APR* isoforms are differently regulated by sulfate and light, and in addition *V. longisporum* infection influences expression either positively or negatively. As was shown by Wang et al. (2011) bacterial infection leads to a reprogramming of the diurnal rhythm and even the circadian clock on expression level. Effects of fungal infections on the genes involved in continuance of diurnal and circadian rhythms need to be further investigated.

With the sulfate assimilation, a co-regulation of the GSL was described (Huseby et al., 2013). GSL act as typical sulfur-containing compounds in Brassicaceae against herbivores and insects occurring in the soil (Halkier and Gershenzon, 2006). Interestingly, experiments done with a fungal pathogen and a Brassicaceae as host generated indication that crude GSL extracts or detached leaf material act defensively (Buxdorf et al., 2013; Witzel et al., 2013). In the *A. thaliana* accessions investigated by Witzel et al. (2013), the total GSL concentrations were about 10 times higher than in our *B. napus* cultivars, where the maximum concentration of total GSL in the leaves was less than 4 μmol g^−1^ TM (data not shown). The most effective GSL with respect to fungal growth inhibition in *A. thaliana* was 2-propenyl GSL that is not present in *B. napus* or can at least not be detected in leaves. In addition, the inhibiting concentration reported by Witzel et al. (2013) of single GSLs would probably not be high enough to reduce the fungal growth rate in our *B. napus* cultivars. Therefore, for the situation in *B. napus* the results by Witzel et al. (2013) are not applicable and relevant. In summary, based on these results and calculations we assume that even if the GSL contents and their composition would differ in the *B. napus* 00 cultivars in high and low sulfur cultivation, we would not see any influence on fungal growth. The role of GSL and their breakdown products in the defense against fungal pathogens needs to be investigated in more detail *in vivo*.

The sulfur-containing glutathione is an important stress indicating metabolite. There are numerous examples that oxidative stress reduces the overall concentration of glutathione, but particularly the concentration of reduced glutathione (GSH). One would expect that after a pathogen attack, the concentration of total glutathione and GSH is drastically reduced. However, there are several examples that this is not the case. For example, an increase of the cysteine and glutathione content was measured in field grown *B. napus* plants due to infection with *Pyrenopeziza brassicae* (Bloem et al., 2004). There is a reduction in GSH observed, but it is not as drastic as one would expect from abiotic stress effects (Cooper et al., 1996; Bloem et al., 2004; Bollig et al., 2013). These results are in agreement with our results. Furthermore, the cysteine content was measured. As a precursor of defense compounds (Smith and Kirkegaard, 2002; Van Wees et al., 2003; Rausch and Wachter, 2005), a decrease of the cysteine content in infected plants is expected. According to results of infected *A. thaliana* plants (Álvarez et al., 2011), a decrease of about 25% in the cysteine content was measured. In comparison to our results, this observation is not supported. In contrast, the content seems to be slightly increased in infected plants. Fluctuations in the content over the day for glutathione and cysteine make an evaluation even more difficult. In conclusion, our results show that the cysteine and glutathione content is more influenced and increased by the light conditions than by pathogen attack.

In experiments of Blake-Kalff et al. (1998), the glutathione concentration decreased in plants when grown under sulfur deficiency during the whole course of the experiment and the glutathione content decreased more rapidly grown on either 0.02 or 0.100 mM sulfate. In leaves of plants grown under sulfur limitation, the sulfur content was strongly negatively affected. Alternatively, the sulfur content increased in roots. This could also be observed under full sulfur supply. About 50% of the energy obtained by photosynthetic activity is transported into the *B. napus* root systems (Agrios, 2005).

### Which cultivar performed best under sulfur limitation and pathogen infection? recommendation for choosing a robust cultivar

The phosphorus contents were always higher in infected plants (about 15%). The results indicate that phosphorus, as one of the macronutrients of the plants with 0.2–0.6% of DM and necessary for the energy metabolism in organisms, plays an important role in pathogen defense, probably due to increased energy demand. Based on the fact that increased phosphorus or nitrogen concentrations have also been reported in other *Verticillium*-infected plants like tomato and *Arabidopsis*, the authors suggested that there is a yet unknown interference of *Verticillium* with the phosphorous or nitrogen metabolism (Floerl et al., 2008). High phosphorus amounts could lead to a decrease in disease but also to an increase in disease after fungal infection (Prabhu et al., 2007). The influence of phosphorus remains unexplained, therefore, high phosphorus values, especially in Exocet after infection, are not discussible.

Iron, essential for biological activity of many proteins mediating electron transfer and redox reactions, is influenced due to infection. Experiments with different pathogens and crop plants revealed an influence of the iron status on the host-pathogen relationships in different ways by affecting the pathogen's virulence as well as the host's defense. Arabidopsis plants, infected with *Erwinia chrysanthemi*, developed an iron-withholding response that involved a change of the iron distribution and trafficking (summarized in Expert et al., 2012). The infection could lead to the release of root exudates for iron mobilization. These observations could be also an explanation for our results especially for the line King10.

In experiments done by D'Hooghe et al. (2013) 2-month-old oilseed rape plants were transferred to sulfur limiting conditions. In these experiments, the total shoot biomass was not significantly reduced, but the growth and the photosynthesis rate were inhibited. The increase of the anthocyanins and the H_2_O_2_ content in sulfur insufficient plants were explained with means of oxidative stress. No significant differences in the chlorophyll and flavonol contents were detectable (D'Hooghe et al., 2013). Prabhu et al. (2007) described that fungistatic substances like phenolic compounds and flavonoids accumulate in epidermal cells of leaves, stems, and roots after infection. We could observe this phenomenon only in line King10.

The accumulation of phytoalexins settled 48 h after inoculation and was accompanied by a more rapid increase in the rate of anthocyanin accumulation. The results suggest that the plant represses less essential metabolic activities, such as anthocyanin synthesis, in order to compensate the immediate biochemical and physiological needs for the defense response (Lo and Nicholson, 1998). According to our results King10 seems to be unaffected by sulfur limitation and infection.

Oilseed rape hybrids have up to 10% higher yields than conventional lines such as King10. The first hybrid cultivars were market-launched in Germany in 1995. Since that time, there has been a discussion which plant performs best: hybrids or bred-lines (Alpmann, 2009). Actually, in our experiments under highly controlled conditions, the hybrid Genie and the line King10 performed equally well with respect to all conditions chosen: to sulfur limitation, reaction to infection, and uptake of nutrients (Figure S1).

In 2010, the cultivars Compass and King10 were mentioned as best performing cultivars with high oil contents (DSV). The oil content has a major influence on the market performance, which depends on the cultivar. Cultivars with high oil content are more attractive with increasing prices for oilseed rape. Already one percent more oil content is paid with a 1.5% higher fee (Alpmann, 2009). Therefore, already in early phases of development general plant fitness and plant health need to be carefully checked to obtain finally high seed yields. In the future, the performance in later phases of the development and finally the seed yield, oil content, and composition need to be studied. Then a recommendation with respect to moderate sulfur limitations and pathogens such as *V. longisporum* could be given. In conclusion, our results indicate that line King10 is the most promising cultivar: Under sulfur deficiency and after infection, line King10 had higher contents of flavonoids and accumulated more sulfur. However, one has to keep in mind that the determination of the biomass of 3-weeks-old plants may not have any correlation to the final yield but early stage changes could be predictive of the further development and the following loss in yield.

### Conflict of interest statement

The authors declare that the research was conducted in the absence of any commercial or financial relationships that could be construed as a potential conflict of interest.

## Footnotes

^1^European Commission Eurostat (2014). Available online at: epp.eurostat.ec.europa.eu/tgm/table.do?tab=table&init=1&language=de&pcode=tag00099&plugin=1. accessed 15.09.2014

^2^Deutsche Saatveredelung, A. G. (2014). Available online at: http://www.dsv-saaten.de/raps/winterraps/sorten [Accessed 01. 07. 2014]

^3^Rapool (2014). Available online at: www.rapool.de [Accessed 01. 07. 2014]

^4^Roth-Agrar (2014). Available online at: roth-agrar.de/produkte/saatgut/saatraps/sortenliste/sortenliste_saatraps_herbst_2013 [Accessed 01. 07. 2014]

^5^Gelanalyzer (2014). Available online at: GelAnalyzer.com [Accessed 16. 09. 2014]

## Supplementary material

Figure S1 **Measurement of iron and phosphorus by ICP-OES**. The elements iron **(A)** and phosphorus **(B)** were measured in dried plant material by ICP-OES. Dried material from plants with five leaves fully expanded and treated as described for Figure 5 were used. Data calculated as mg g^−1^ DM represent the mean of three dependently technical replicates ± SD. Filled dots represent the mock-inoculated plants **(C)** and open dots show the corresponding results of infected plants (INF).

Figure S2 **Total flavonoids**. The total flavonoid content was photometrically measured in the cultivars with five fully expanded leaves treated as described for Figure 5. The results are given in μg g^.−1^ FM as mean of three technical replicates ± SD. For quantification catechin was used as a standard. Filled bars represent the control plants (C) for different sulfur fertilization and striped bars indicate infected plants (INF). CE, catechin equivalent.

Figure S3 **Expression analysis**. Results of Northern Blot analysis (Figure 10) were evaluated with the program GelAnalyzer (GelAnalyzer.com). Intensity for each band detected on the membrane was calculated in reference to the first sample (0 h). Data represent the relative expression in %. Values of non-infected plants with 1 mM MgSO_4_ at 0 h were used as 100% for control and infected plants for each cultivar. **(A)** *SULTR4;2*, **(B)** *APR2*, and **(C)** *APR3*. Filled dots represent the mock-inoculated plants (C) and open dots show the corresponding results of infected plants (INF).
