# Supplementary material for: Brassica napus L. cultivars show a broad variability in their morphology, physiology and metabolite levels in response to sulfur limitations and to pathogen attack
Source: Front Plant Sci. 2015 Feb 2;6:9. doi: 10.3389/fpls.2015.00009 (PMC4313603; doi:10.3389/fpls.2015.00009)
Supplement: Figure S1 — Measurement of iron and phosphorus by ICP-OES. The elements iron (A) and phosphorus (B) were measured in dried plant material by ICP-OES. Dried material from plants with five leaves fully expanded and treated as described for Figure 5 were used. Data calculated as mg g−1 DM represent the mean of three dependently technical replicates ± SD. Filled dots represent the mock-inoculated plants (C) and open dots show the corresponding results of infected plants (INF). [file DataSheet1.DOCX]

**Supplementary Information**

**
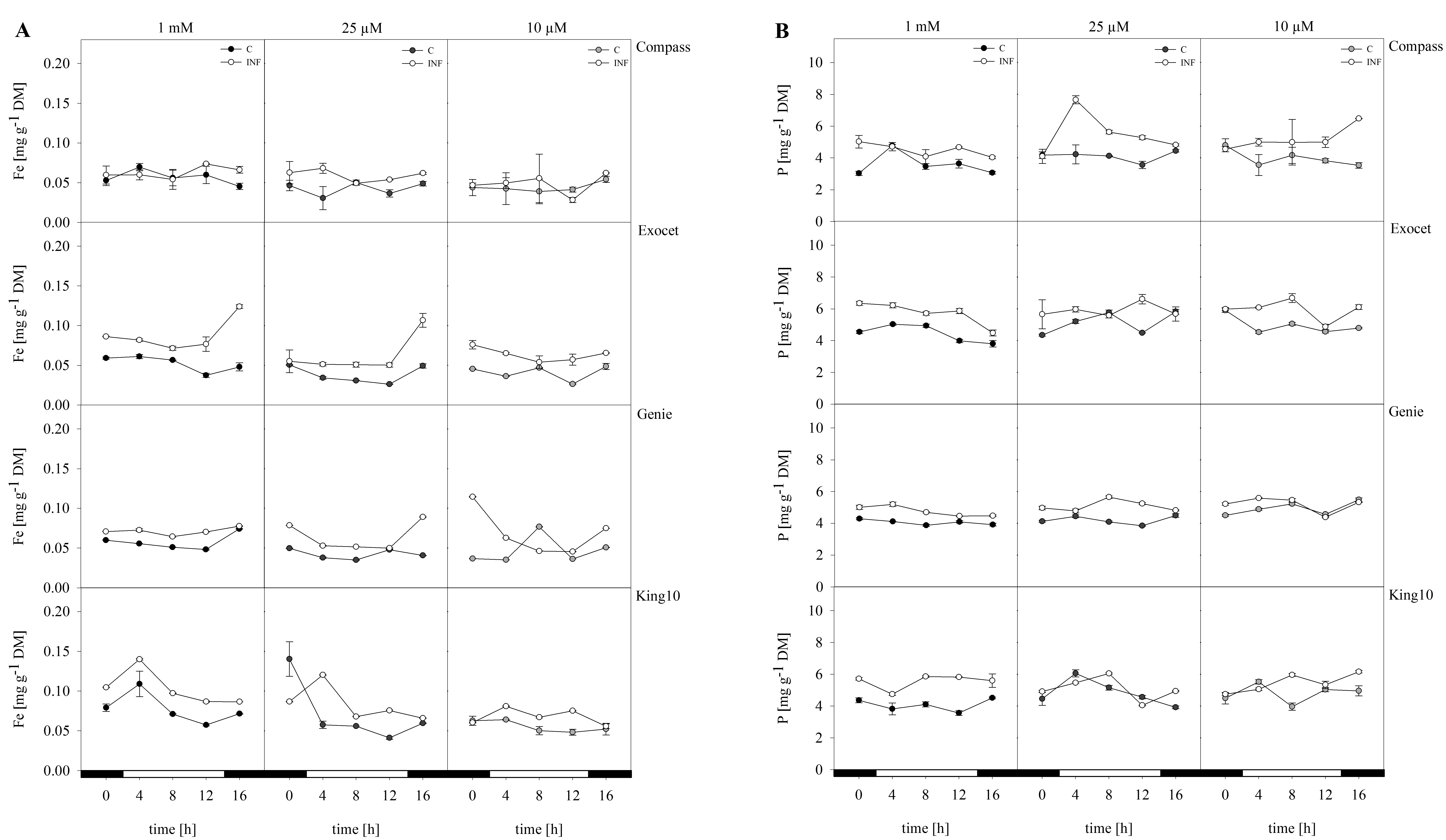
Figure S1.**

**Figure S2.**


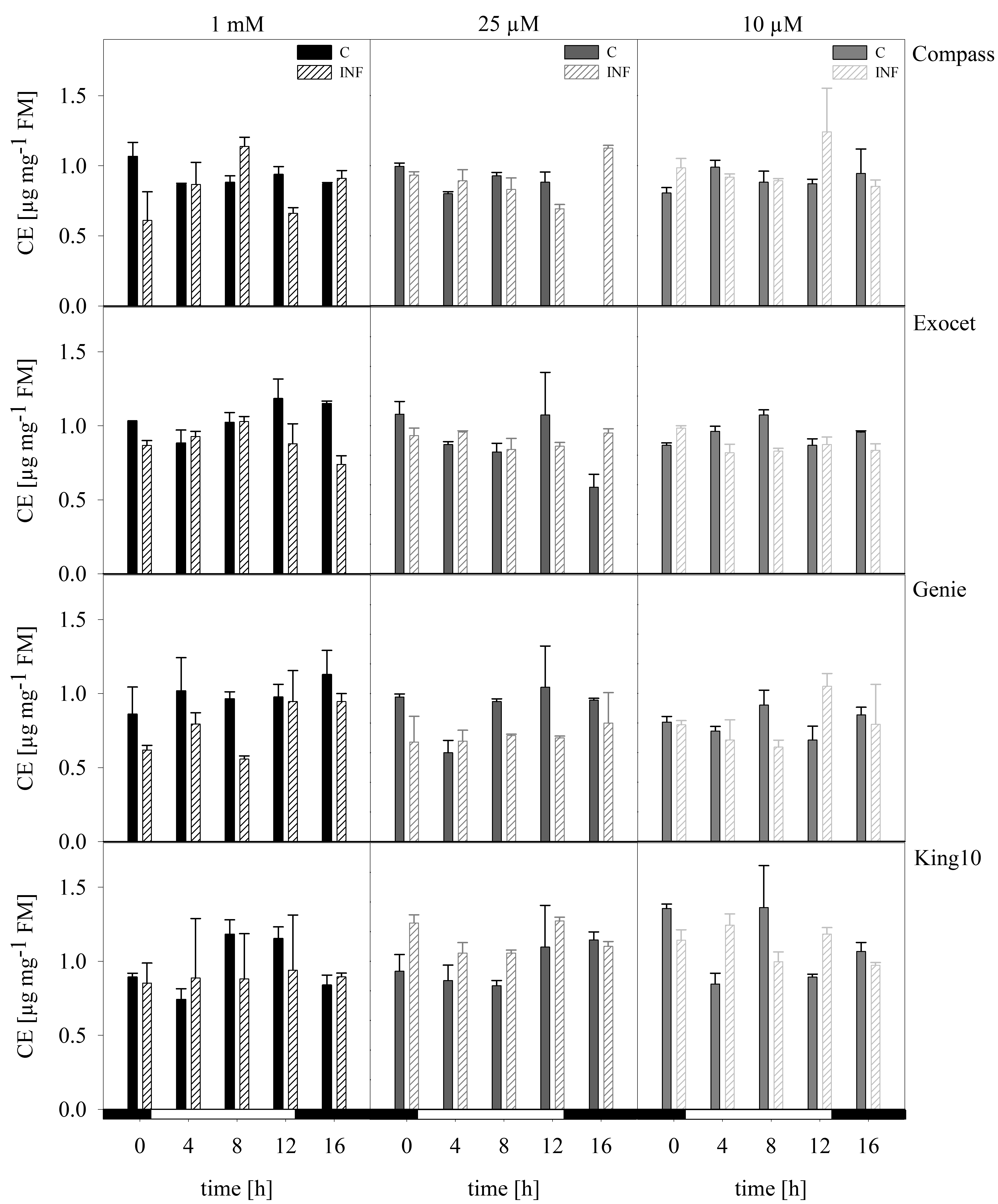


**Figure S3.** **
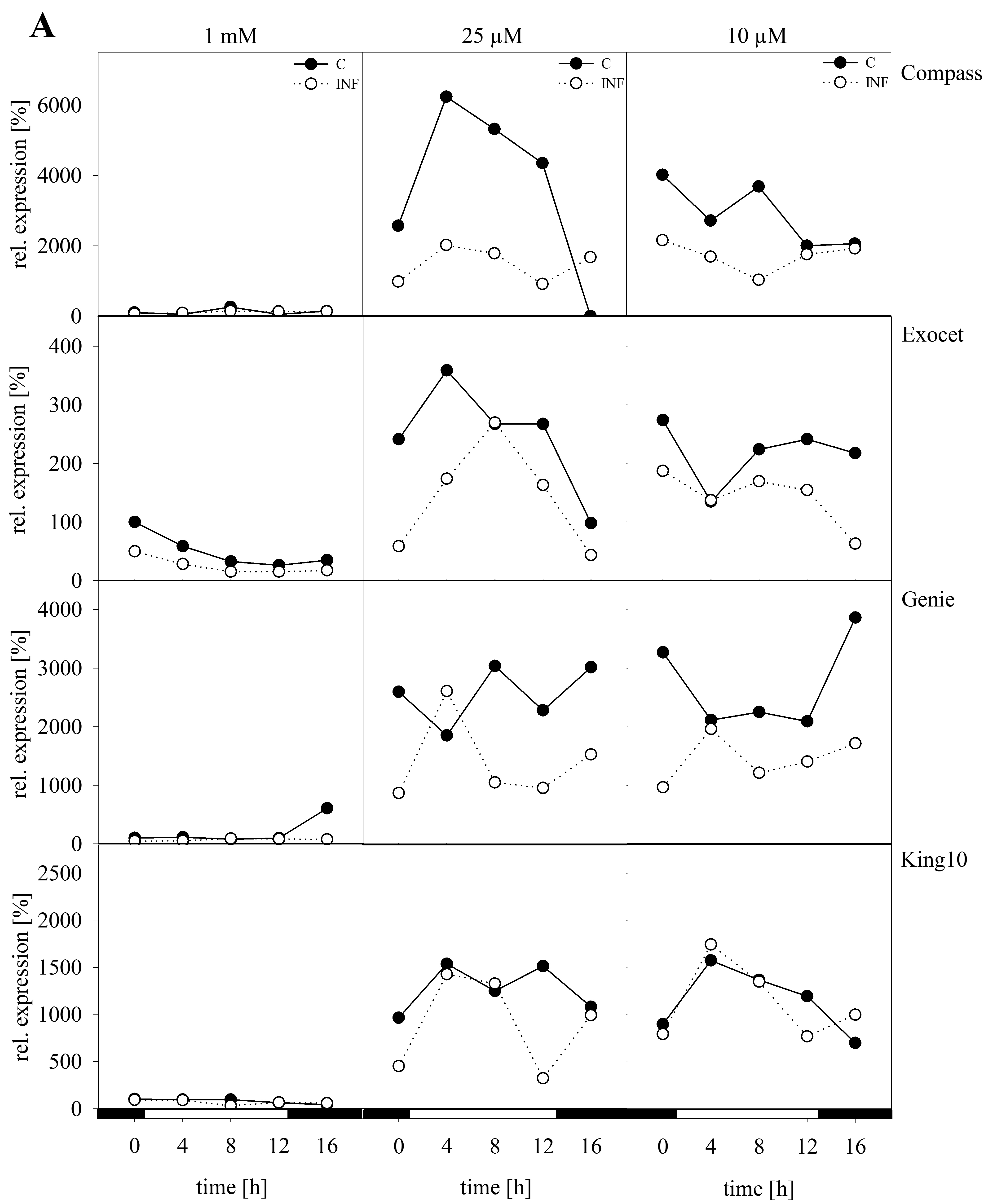
**

**
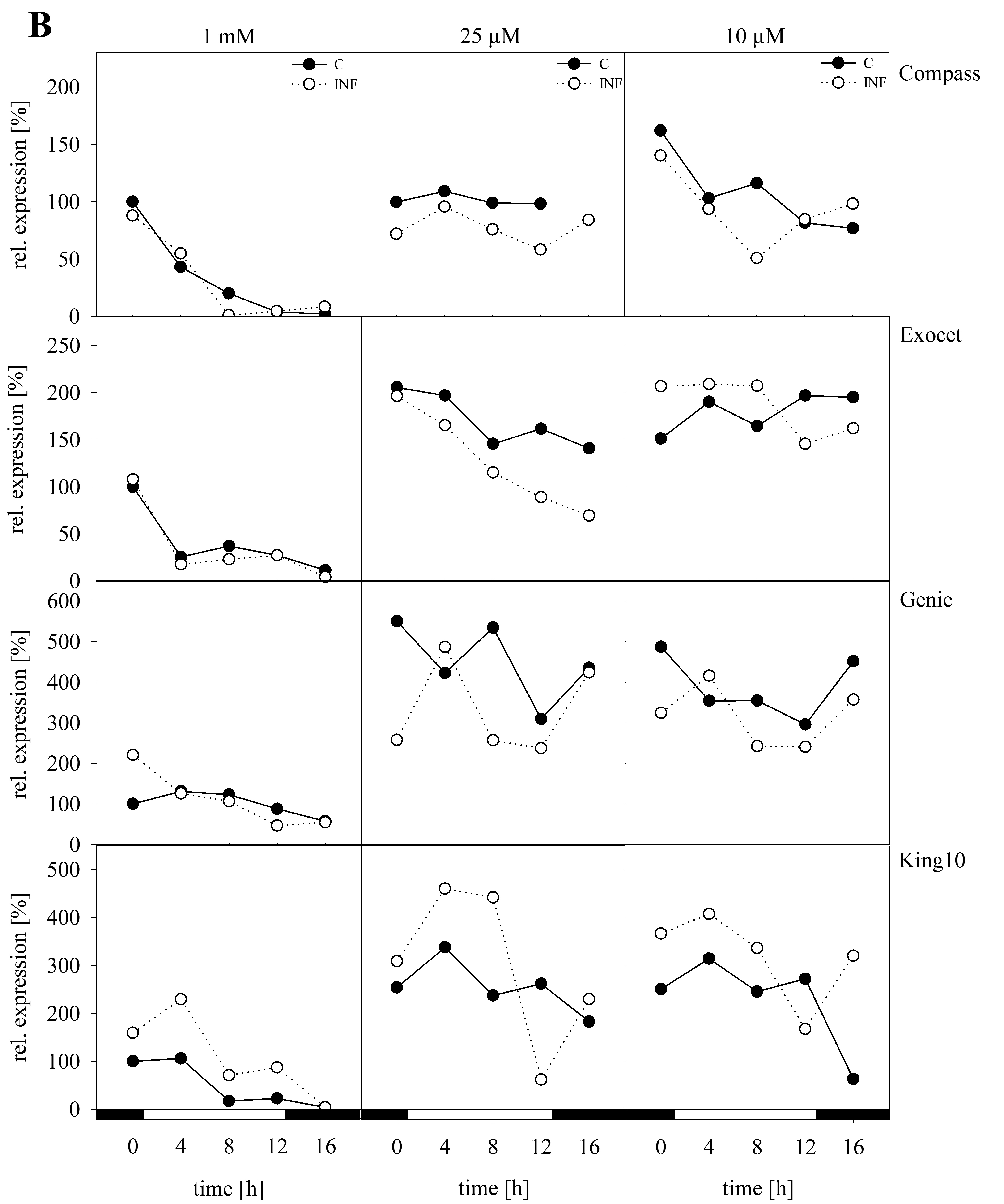

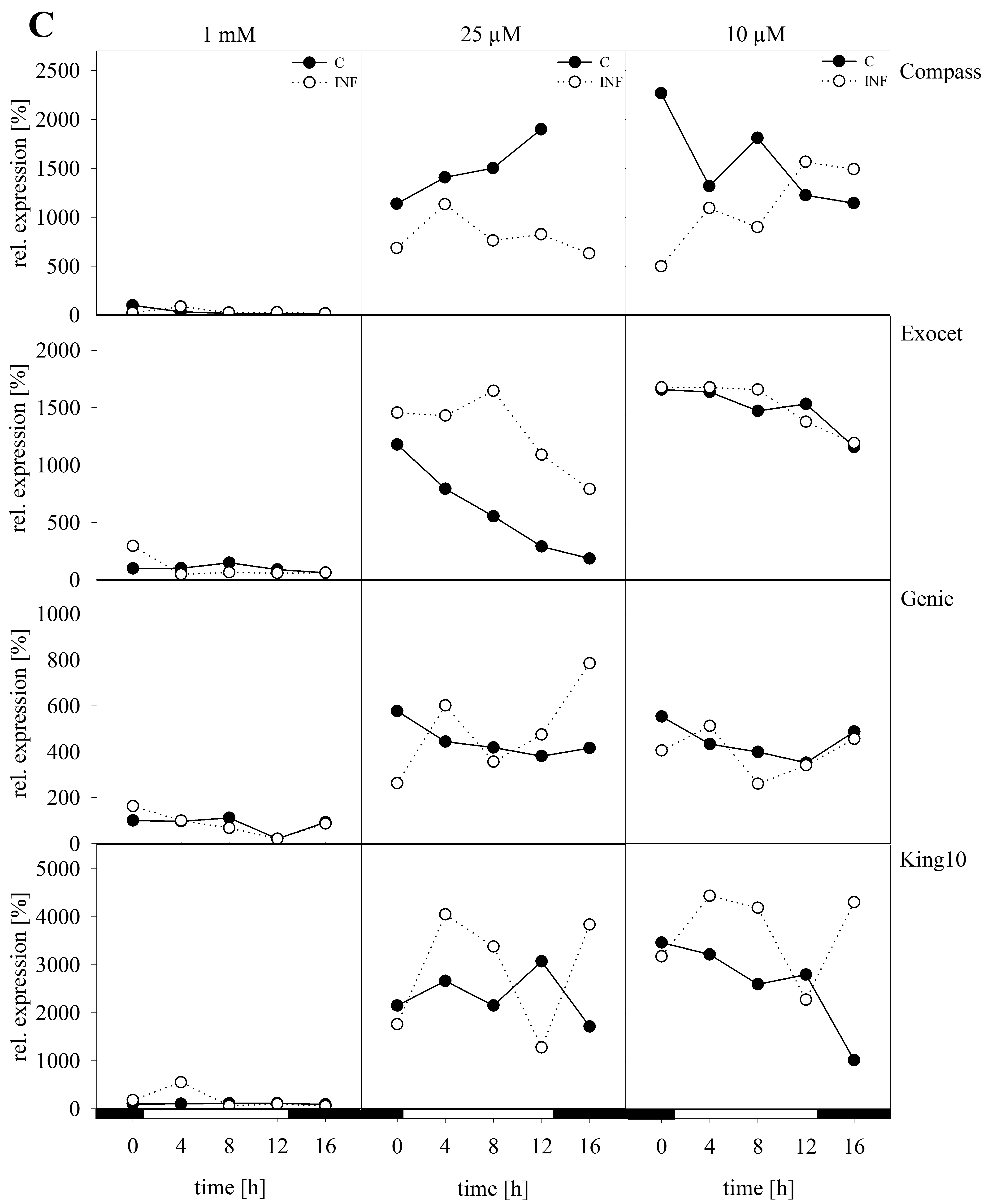
**

**Table S1. Differences in root hair length among the varieties.** Statistical tests were performed using Sigma Plot. Mean values of five plants ± SD are shown along with letters indicating significant differences for pairwise comparisons (Tukey test) among the cultivars.

|  | **Root hair length [cm]** | |  |
| --- | --- | --- | --- |
| Compass | Exocet | Genie | King10 |
|  |  |  |  |
| 1.166±0.282^a^ | 1.14±0.287^a^ | 0.582±0.079^b^ | 0.698±0.085^b^ |

**Table S2. Influence of S-fertilization, infection and cultivar on the total dry mass.** Statistical analysis was performed using R. The p-values refer to pairwise comparisons (Tukey test) of the data shown in Figure 2A. Significances: p<0.05*, p<0.01**,p<0.001***.

| **variable** | **comparison** | **p-value** | **significance** |
| --- | --- | --- | --- |
| S-fertilization | 1 mM – 0.025 mM | <0.001 | $***$ |
|  | 1 mM – 0.01 mM | <0.001 | $***$ |
|  | 0.025 mM – 0.01 mM | 0.556 | ns |
| infection | Compass: INF - C | 0.064 | ns |
|  | Exocet: INF - C | <0.001 | $***$ |
|  | Genie: INF - C | <0.001 | $***$ |
|  | King10: INF - C | 0.100 | ns |
| cultivar | C: Compass - Exocet | 0.639 | ns |
|  | C: Compass - Genie | 0.015 | $*$ |
|  | C: Compass - King10 | <0.001 | $***$ |
|  | C: Exocet - Genie | 0.260 | ns |
|  | C: Exocet - King10 | <0.001 | $**$ |
|  | C: Genie - King10 | 0.546 | ns |
|  | INF: Compass - Exocet | <0.001 | $***$ |
|  | INF: Compass - Genie | <0.001 | $***$ |
|  | INF: Compass - King10 | <0.001 | $***$ |
|  | INF: Exocet - Genie | 0.367 | ns |
|  | INF: Exocet - King10 | 0.989 | ns |
|  | INF: Genie - King10 | 0.206 | ns |

**Table S3. Influence of S-fertilization, infection and cultivar on the shoot-to-root ratio.** Statistical tests were performed using R. The p-values refer to pairwise comparisons (Tukey tests) of the data shown in Figure 2B. Significances: p<0.05*, p<0.01**, p<0.001***.

| **variable** | **comparison** | **p-value** | **significance** |
| --- | --- | --- | --- |
| S-fertilization | 1 mM – 0.025 mM | <0.001 | $**$ |
|  | 1 mM – 0.010 mM | 0.082 | ns |
|  | 0.025 mM – 0.010 mM | 0.510 | ns |
| infection | INF- C | 0.220 | ns |
| cultivar | Compass - Exocet | <0.001 | $***$ |
|  | Compass - Genie | <0.001 | $***$ |
|  | Compass - King10 | <0.001 | $***$ |
|  | Exocet - Genie | 0.918 | ns |
|  | Exocet - King10 | 0.033 | $*$ |
|  | Genie - King10 | 0.154 | ns |

**Table S4. Influence of the interaction of S-fertilization, infection and cultivar on the leaf temperature.** Statistical analysis was performed using R. The p-values refer to pairwise comparisons (Tukey tests) of the data shown in Figure 3. Since all interactions were significant in the F-test, comparisons were carried out separately at all factor levels. Significances: p<0.05*, p<0.01**, p<0.001***.

| **variable** | **comparison** | **p-value** | **significance** |
| --- | --- | --- | --- |
| S-fertilization | C: Compass: 1 mM – 0.025 mM | 0.413 | ns |
|  | C: Compass: 1 mM – 0.01 mM | 0.999 | ns |
|  | C: Compass: 0.025 mM – 0.01 mM | 0.387 | ns |
|  | C: Exocet: 1 mM - 0.025 mM | 0.858 | ns |
|  | C: Exocet: 1 mM - 0.01 mM | 0.747 | ns |
|  | C: Exocet: 0.025 mM - 0.01 mM | 0.422 | ns |
|  | C: Genie: 1 mM - 0.025 mM | 0.945 | ns |
|  | C: Genie: 1 mM - 0.01 mM | 0.122 | ns |
|  | C: Genie: 0.025 mM - 0.01 mM | 0.058 | ns |
|  | C: King10: 1 mM - 0.025 mM | 0.339 | ns |
|  | C: King10: 1 mM - 0.01 mM | 0.859 | ns |
|  | C: King10: 0.025 mM - 0.01 mM | 0.654 | ns |
|  | INF: Compass: 1 mM - 0.025 mM | <0.001 | $***$ |
|  | INF: Compass: 1 mM - 0.01 mM | 0.729 | ns |
|  | INF: Compass: 0.025 mM - 0.01 mM | <0.001 | $***$ |
|  | INF: Exocet: 1 mM - 0.025 mM | 0.673 | ns |
|  | INF: Exocet: 1 mM - 0.01 mM | 0.686 | ns |
|  | INF: Exocet: 0.025 mM - 0.01 mM | 0.999 | ns |
|  | INF: Genie: 1 mM - 0.025 mM | 0.851 | ns |
|  | INF: Genie: 1 mM - 0.01 mM | 0.971 | ns |
|  | INF: Genie: 0.025 mM - 0.01 mM | 0.948 | ns |
|  | INF: King10: 1 mM - 0.025 mM | <0.001 | $***$ |
|  | INF: King10: 1 mM - 0.01 mM | 0.004 | $**$ |
|  | INF: King10: 0.025 mM - 0.01 mM | 0.006 | $**$ |
| infection | 1 mM: Compass: INF - C | 0.869 | ns |
|  | 1 mM: Exocet: INF - C | 0.251 | ns |
|  | 1 mM: Genie: INF - C | 0.537 | ns |
|  | 1 mM: King10: INF - C | 0.516 | ns |
|  | 0.025 mM: Compass: INF - C | <0.001 | $***$ |
|  | 0.025 mM: Exocet: INF - C | 0.012 | $*$ |
|  | 0.025 mM: Genie: INF - C | 0.402 | ns |
|  | 0.025 mM: King10: INF - C | <0.001 | $***$ |
|  | 0.01 mM: Compass: INF - C | 0.333 | ns |
|  | 0.01 mM: Exocet: INF - C | 0.212 | ns |
|  | 0.01 mM: Genie: INF - C | 0.005 | $**$ |
|  | 0.01 mM: King10: INF - C | <0.001 | $***$ |
| cultivar | C: 1 mM: Compass - Exocet | 0.999 | ns |
|  | C: 1 mM: Compass - Genie | 0.573 | ns |
|  | C: 1 mM: Compass - King10 | 0.675 | ns |
|  | C: 1 mM: Exocet - Genie | 0.637 | ns |
|  | C: 1 mM: Exocet - King10 | 0.736 | ns |
|  | C: 1 mM: Genie - King10 | 0.999 | ns |
|  | C: 0.025 mM: Compass - Exocet | 0.326 | ns |
|  | C: 0.025 mM: Compass - Genie | 0.987 | ns |
|  | C: 0.025 mM: Compass - King10 | 0.589 | ns |
|  | C: 0.025 mM: Exocet - Genie | 0.177 | ns |
|  | C: 0.025 mM: Exocet - King10 | 0.016 | $*$ |
|  | C: 0.025 mM: Genie - King10 | 0.793 | ns |
|  | C: 0.01 mM: Compass - Exocet | 0.819 | ns |
|  | C: 0.01 mM: Compass - Genie | 0.922 | ns |
|  | C: 0.01 mM: Compass - King10 | 0.326 | ns |
|  | C: 0.01 mM: Exocet - Genie | 0.435 | ns |
|  | C: 0.01 mM: Exocet - King10 | 0.844 | ns |
|  | C: 0.01 mM: Genie - King10 | 0.092 | ns |
|  | INF: 1 mM: Compass - Exocet | 0.700 | ns |
|  | INF: 1 mM: Compass - Genie | 0.305 | ns |
|  | INF: 1 mM: Compass - King10 | 0.374 | ns |
|  | INF: 1 mM: Exocet - Genie | 0.915 | ns |
|  | INF: 1 mM: Exocet - King10 | 0.953 | ns |
|  | INF: 1 mM: Genie - King10 | 0.999 | ns |
|  | INF: 0.025 mM: Compass - Exocet | <0.001 | $***$ |
|  | INF: 0.025 mM: Compass - Genie | <0.001 | $***$ |
|  | INF: 0.025 mM: Compass - King10 | 0.816 | ns |
|  | INF: 0.025 mM: Exocet - Genie | 0.986 | ns |
|  | INF: 0.025 mM: Exocet - King10 | <0.001 | $***$ |
|  | INF: 0.025 mM: Genie - King10 | <0.001 | $***$ |
|  | INF: 0.01 mM: Compass - Exocet | 0.656 | ns |
|  | INF: 0.01 mM: Compass - Genie | 0.621 | ns |
|  | INF: 0.01 mM: Compass - King10 | <0.001 | $***$ |
|  | INF: 0.01 mM: Exocet - Genie | 0.999 | ns |
|  | INF: 0.01 mM: Exocet - King10 | 0.019 | $*$ |
|  | INF: 0.01 mM: Genie - King10 | 0.022 | $*$ |

**Table S5**. **Influence of the interaction of S-fertilization, infection and cultivar on the cholorophyll fluorescence.** Statistical analysis was performed using R. The p-values refer to pairwise comparisons (Tukey test) of the data shown in Figure 4. Since all the interactions were significant in the F-test, comparisons were carried out separately at all factor levels. Significances: p<0.05*, p<0.01**, p<0.001***.

| **variable** | **comparison** | **p-value** | **significance** |
| --- | --- | --- | --- |
| S-fertilization | C: Compass: 1 mM - 0.025 mM | <0.001 | $***$ |
|  | C: Compass: 1 mM - 0.01 mM | <0.001 | $***$ |
|  | C: Compass: 0.025 mM - 0.01 mM | 0.633 | ns |
|  | C: Exocet: 1 mM - 0.025 mM | <0.001 | $***$ |
|  | C: Exocet: 1 mM - 0.01 mM | 0.999 | ns |
|  | C: Exocet: 0.025 mM - 0.01 mM | <0.001 | $***$ |
|  | C: Genie: 1 mM - 0.025 mM | 0.964 | ns |
|  | C: Genie: 1 mM - 0.01 mM | 0.861 | ns |
|  | C: Genie: 0.025 mM - 0.01 mM | 0.962 | ns |
|  | C: King0.01: 1 mM - 0.025 mM | <0.001 | $***$ |
|  | C: King0.01: 1 mM - 0.01 mM | <0.001 | $***$ |
|  | C: King0.01: 0.025 mM - 0.01 mM | 0.569 | ns |
|  | Inf: Compass: 1 mM - 0.025 mM | <0.001 | $***$ |
|  | Inf: Compass: 1 mM - 0.01 mM | <0.001 | $***$ |
|  | Inf: Compass: 0.025 mM - 0.01 mM | 0.964 | ns |
|  | Inf: Exocet: 1 mM - 0.025 mM | <0.001 | $***$ |
|  | Inf: Exocet: 1 mM - 0.01 mM | <0.001 | $***$ |
|  | Inf: Exocet: 0.025 mM - 0.01 mM | 0.500 | ns |
|  | Inf: Genie: 1 mM - 0.025 mM | <0.001 | $***$ |
|  | Inf: Genie: 1 mM - 0.01 mM | <0.001 | $***$ |
|  | Inf: Genie: 0.025 mM - 0.01 mM | 0.781 | ns |
|  | Inf: King0.01: 1 mM - 0.025 mM | <0.001 | $***$ |
|  | Inf: King0.01: 1 mM - 0.01 mM | <0.001 | $***$ |
|  | Inf: King0.01: 0.025 mM - 0.01 mM | 0.845 | ns |
| infection | 1 mM: Compass: Inf - C | 0.474 | ns |
|  | 1 mM: Exocet: Inf - C | 0.360 | ns |
|  | 1 mM: Genie: Inf - C | 0.681 | ns |
|  | 1 mM: King0.01: Inf - C | 0.706 | ns |
|  | 0.025 mM: Compass: Inf - C | <0.001 | $***$ |
|  | 0.025 mM: Exocet: Inf - C | 0.001 | $**$ |
|  | 0.025 mM: Genie: Inf - C | <0.001 | $***$ |
|  | 0.025 mM: King0.01: Inf - C | 0.896 | ns |
|  | 0.01 mM: Compass: Inf - C | 0.030 | $*$ |
|  | 0.01 mM: Exocet: Inf - C | <0.001 | $***$ |
|  | 0.01 mM: Genie: Inf - C | <0.001 | $***$ |
|  | 0.01 mM: King0.01: Inf - C | 0.555 | ns |
| cultivar | C: 1 mM: Compass - Exocet | 0.812 | ns |
|  | C: 1 mM: Compass - Genie | 0.702 | ns |
|  | C: 1 mM: Compass - King0.01 | 0.997 | ns |
|  | C: 1 mM: Exocet - Genie | 0.997 | ns |
|  | C: 1 mM: Exocet - King0.01 | 0.899 | ns |
|  | C: 1 mM: Genie - King0.01 | 0.812 | ns |
|  | C: 0.025 mM: Compass - Exocet | 0.748 | ns |
|  | C: 0.025 mM: Compass - Genie | 0.091 | ns |
|  | C: 0.025 mM: Compass - King0.01 | 0.002 | $**$ |
|  | C: 0.025 mM: Exocet - Genie | 0.005 | $**$ |
|  | C: 0.025 mM: Exocet - King0.01 | 0.044 | $*$ |
|  | C: 0.025 mM: Genie - King0.01 | 0. | $***$ |
|  | C: 0.01 mM: Compass - Exocet | 0.001 | $**$ |
|  | C: 0.01 mM: Compass - Genie | 0.016 | $*$ |
|  | C: 0.01 mM: Compass - King0.01 | 0.324 | ns |
|  | C: 0.01 mM: Exocet - Genie | 0.894 | ns |
|  | C: 0.01 mM: Exocet - King0.01 | 0. | $***$ |
|  | C: 0.01 mM: Genie - King0.01 | 0. | $***$ |
|  | Inf: 1 mM: Compass - Exocet | 0.698 | ns |
|  | Inf: 1 mM: Compass - Genie | 0.866 | ns |
|  | Inf: 1 mM: Compass - King0.01 | 0.999 | ns |
|  | Inf: 1 mM: Exocet - Genie | 0.989 | ns |
|  | Inf: 1 mM: Exocet - King0.01 | 0.606 | ns |
|  | Inf: 1 mM: Genie - King0.01 | 0.794 | ns |
|  | Inf: 0.025 mM: Compass - Exocet | 0.817 | ns |
|  | Inf: 0.025 mM: Compass - Genie | 0.961 | ns |
|  | Inf: 0.025 mM: Compass - King0.01 | 0.976 | ns |
|  | Inf: 0.025 mM: Exocet - Genie | 0.519 | ns |
|  | Inf: 0.025 mM: Exocet - King0.01 | 0.967 | ns |
|  | Inf: 0.025 mM: Genie - King0.01 | 0.802 | ns |
|  | Inf: 0.01 mM: Compass - Exocet | 0.108 | ns |
|  | Inf: 0.01 mM: Compass - Genie | 0.972 | ns |
|  | Inf: 0.01 mM: Compass - King0.01 | 0.999 | ns |
|  | Inf: 0.01 mM: Exocet - Genie | 0.263 | ns |
|  | Inf: 0.01 mM: Exocet - King0.01 | 0.141 | ns |
|  | Inf: 0.01 mM: Genie - King0.01 | 0.989 | ns |
